# In situ vaccination via tissue-targeted cDC1 expansion enhances the immunogenicity of chemoradiation and immunotherapy

**DOI:** 10.1172/JCI171621

**Published:** 2024-01-02

**Authors:** Brandon Lam, Yu Jui Kung, John Lin, Ssu-Hsueh Tseng, Hsin-Fang Tu, Claire Huang, Brandon Lee, Esteban Velarde, Ya Chea Tsai, Rafael Villasmil, Sung Taek Park, Deyin Xing, Chien-Fu Hung, T.-C. Wu

**Affiliations:** 1 Department of Pathology and; 2 Graduate Program in Immunology, Johns Hopkins University School of Medicine, Baltimore, Maryland, USA.; 3 Stanford Medicine, Stanford University School of Medicine, Stanford, California, USA.; 4 Department of Radiation Oncology and Molecular Sciences, Johns Hopkins University School of Medicine, Baltimore, Maryland, USA.; 5 Laboratory of Immunology, National Eye Institute, NIH, Bethesda, Maryland, USA.; 6 Department of Obstetrics and Gynecology, Hallym University Kangnam Sacred Heart Hospital, Seoul, South Korea.; 7 Department of Oncology,; 8 Department of Obstetrics and Gynecology,; 9 Molecular Microbiology and Immunology, Johns Hopkins University School of Medicine, Baltimore, Maryland, USA.

**Keywords:** Immunology, Vaccines, Adaptive immunity, Cancer immunotherapy, Dendritic cells

## Abstract

Even with the prolific clinical use of next-generation cancer therapeutics, many tumors remain unresponsive or become refractory to therapy, creating a medical need. In cancer, DCs are indispensable for T cell activation, so there is a restriction on cytotoxic T cell immunity if DCs are not present in sufficient numbers in the tumor and draining lymph nodes to take up and present relevant cancer antigens. To address this bottleneck, we developed a therapeutic based on albumin fused with FMS-related tyrosine kinase 3 ligand (Alb-Flt3L) that demonstrated superior pharmacokinetic properties compared with Flt3L, including significantly longer half-life, accumulation in tumors and lymph nodes, and cross-presenting-DC expansion following a single injection. We demonstrated that Alb-Flt3L, in combination with standard-of-care chemotherapy and radiation therapy, serves as an in situ vaccination strategy capable of engendering polyclonal tumor neoantigen–specific immunity spontaneously. In addition, Alb-Flt3L–mediated tumor control synergized with immune checkpoint blockade delivered as anti–PD-L1. The mechanism of action of Alb-Flt3L treatment revealed a dependency on Batf3, type I IFNs, and plasmacytoid DCs. Finally, the ability of Alb-Flt3L to expand human DCs was explored in humanized mice. We observed significant expansion of human cross-presenting-DC subsets, supporting the notion that Alb-Flt3L could be used clinically to modulate human DC populations in future cancer therapeutic regimens.

## Abstract 2

Alb-Flt3L immunotherapeutic molecule enhances antitumor immunity and tumor control alongside standard-of-care chemoradiation and checkpoint blockade.

## Graphical abstract

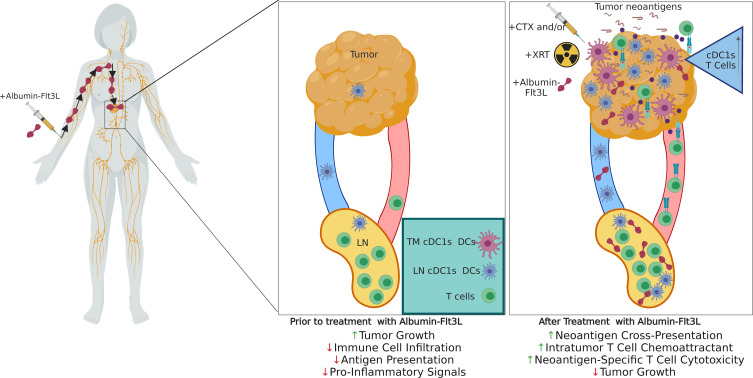

## Introduction

The ability to engender potent neoantigen- or tumor-associated antigen–specific CD8^+^ cytotoxic T cell immunity is indispensable to the long-term therapeutic efficacy of essentially all cancer treatment strategies. Classical methods such as chemotherapy and radiation therapy have been shown to induce cell apoptosis, antigen release, loading on MHC molecules, and finally the activation of tumor-specific CD8^+^ T cells (1). Newer-generation immunotherapies, such as immune checkpoint inhibitors (e.g., PD-1/PD-L1), block receptors involved in T cell suppression or exhaustion, which allows for the “brakes” to be removed from T cells and productive tumor-specific engagement to occur (2, 3).

A plausible approach to circumvent roadblocks in generating tumor-specific CD8^+^ T cell responses against tumor antigens is through the use of cancer vaccines, where tumor antigen is delivered to the host to enhance antigen-specific immunity (4). In next-generation cancer vaccine approaches, exome sequencing combined with RNA-seq is used to identify mutations expressed by the patient’s tumor, and a personalized vaccine containing these neoantigens and tumor-associated antigens can be developed, which hypothetically can directly prime and expand the appropriate tumor-specific T cells (5). Unfortunately, such vaccines currently require a great deal of time from tissue acquisition to vaccine availability (3–5 months), are costly, and must be personalized for each patient (6). These vaccines must also compete with the continually evolving tumor mutational landscape, immunoselection, and immune escape. This is in addition to the fact that antigenicity of tumor-bearing mutations can be very difficult to predict. For example, in HPV-associated cancers, it may be intuitive to think that the generation of HPV-specific CD8^+^ T cell cytotoxicity will lead to tumor regression and control, but this is not necessarily the case. Analyzing the antitumor T cell repertoire in patients with HPV^+^ cervical cancer that underwent regression revealed that tumor-reactive T cell clones were not HPV directed, but instead recognized neoantigens or other nonmutated tumor-associated antigens (7).

As an alternative to cancer vaccines, antigen presentation in the host can be modulated to drive the presentation of tumor antigens and subsequent CD8^+^ T cell immunity. Because tumor cells do not efficiently present immunostimulatory epitopes to CD8^+^ T cells, potentially due to subpar antigen processing or surface downregulation of MHCI, antigen-presenting cells, specifically dendritic cells (DCs) capable of cross-presenting tumor-derived antigens, are often required to elicit potent CD8^+^ T cell immunity. Homeostatically, DCs exist as various subpopulations with different functionality and a great deal of plasticity. Human DCs found in the blood or spleen can be subdivided into 3 major classes: conventional type 1 (cDC1) (CD141^+^), cDC2 (CD1c^+^), and plasmacytoid (pDC) (CD303^+^) (8–10). These DC subsets in the spleen or blood most closely resemble mouse CD8a^+^ DCs, CD11b^+^ DCs, and pDCs, respectively (11). While all of these DC subsets in mouse and human can prime T helper cells (12), only cDC1 or mouse CD8a^+^ DCs (CD103^+^ in tissue) can efficiently take up antigen and cross-prime cytotoxic CD8^+^ T cells. Recent studies have highlighted the importance of CD8a^+^ DCs and tumor-resident CD103^+^ DCs in antitumor immunity due to their ability to secrete chemokines such as CXCL10, which recruit T cells to the tumor, and cytokines such as IL-12 to enhance the type I inflammatory state of infiltrating T cells (13, 14).

Therapeutic strategies have been developed to expand DCs using granulocyte-macrophage colony–stimulating factor (GM-CSF) (15) (e.g., GVAX) (16, 17), but clinical success has been unclear, possibly because GM-CSF does not efficiently expand cross-priming DCs. FMS-like tyrosine kinase 3 ligand (Flt3L) is a cytokine that has been shown to be critical to the survival, expansion, and differentiation of hematopoietic progenitors into DCs (18). Notably, Flt3L can preferentially skew progenitors to cross-presenting-DC subsets (19). Due to the capability of Flt3L to expand cross-presenting-DC populations, Flt3L has been assessed in both healthy and cancer patients as a candidate immunotherapeutic (20, 21), and has been previously incorporated into various therapeutic vaccine designs and treatment strategies (22–28). In this study, we describe the use of an Flt3L-based immunotherapeutic named albumin-Flt3L (Alb-Flt3L) with potent cross-presenting-DC expansion abilities as an in situ vaccination strategy to promote tumor antigen–specific immunity in combination with standard-of-care chemoradiation. The scientific rationale behind combining chemoradiation with Alb-Flt3L is 2-fold. First, due to the induction of cell death and apoptosis, chemoradiation drives the release of tumor antigen, providing more access to antigen for Alb-Flt3L–expanded DCs to take up and cross-present. Secondly, it has been well characterized that chemoradiation acts as an adjuvant to drive potent immunostimulation and potentiation of immunotherapy-driven responses through multiple mechanisms (29–34).

## Results

### Alb-Flt3L has favorable pharmacokinetic properties compared with Flt3L.

A significant drawback to utilizing Flt3L as a cancer therapeutic strategy is its short half-life and disperse distribution pattern in vivo (35). Perhaps this is one of the reasons why Flt3L has been administered daily for 9 days in murine models as a part of other experimental designs in the literature (36). Other groups have tried to address the half-life limitations of Flt3L through various designs (35, 37). Albumin is a ubiquitous protein in the body known for its long half-life in vivo (38) due to binding and transcytolic cycling with the neonatal Fc receptor (FcRn), also known as the Brambell receptor (39). Physiologically, albumin binds to soluble factors as well as antigens in the blood and facilitates trafficking to the appropriate tissues (40). Notably, due to its circulation pattern through the body as a plasma protein, it is drained into lymph nodes (LNs) and as a result, albumin can be utilized to carry cargo preferentially to LNs (40). Furthermore, studies have also shown that albumin binding can promote intratumoral accumulation of molecules such as chemotherapeutic agents to enhance delivery of payload directly to tumor tissue (41).

To harness the potent ability of Flt3L to expand cross-presenting DCs and albumin to extend half-life while preferentially trafficking to the LNs and tumor, we generated an immunotherapeutic molecule, Alb-Flt3L, by genetically fusing albumin to Flt3L (Supplemental Figure 1, A–D; supplemental material available online with this article; https://doi.org/10.1172/JCI171621DS1). Our approach is different from other methods, such as albumin “hitchhiking,” where antigen or adjuvant cargo linked to a lipophilic albumin-binding tail can be used for albumin binding in order to target the LNs to improve immune responses (42).

**Figure 1:**
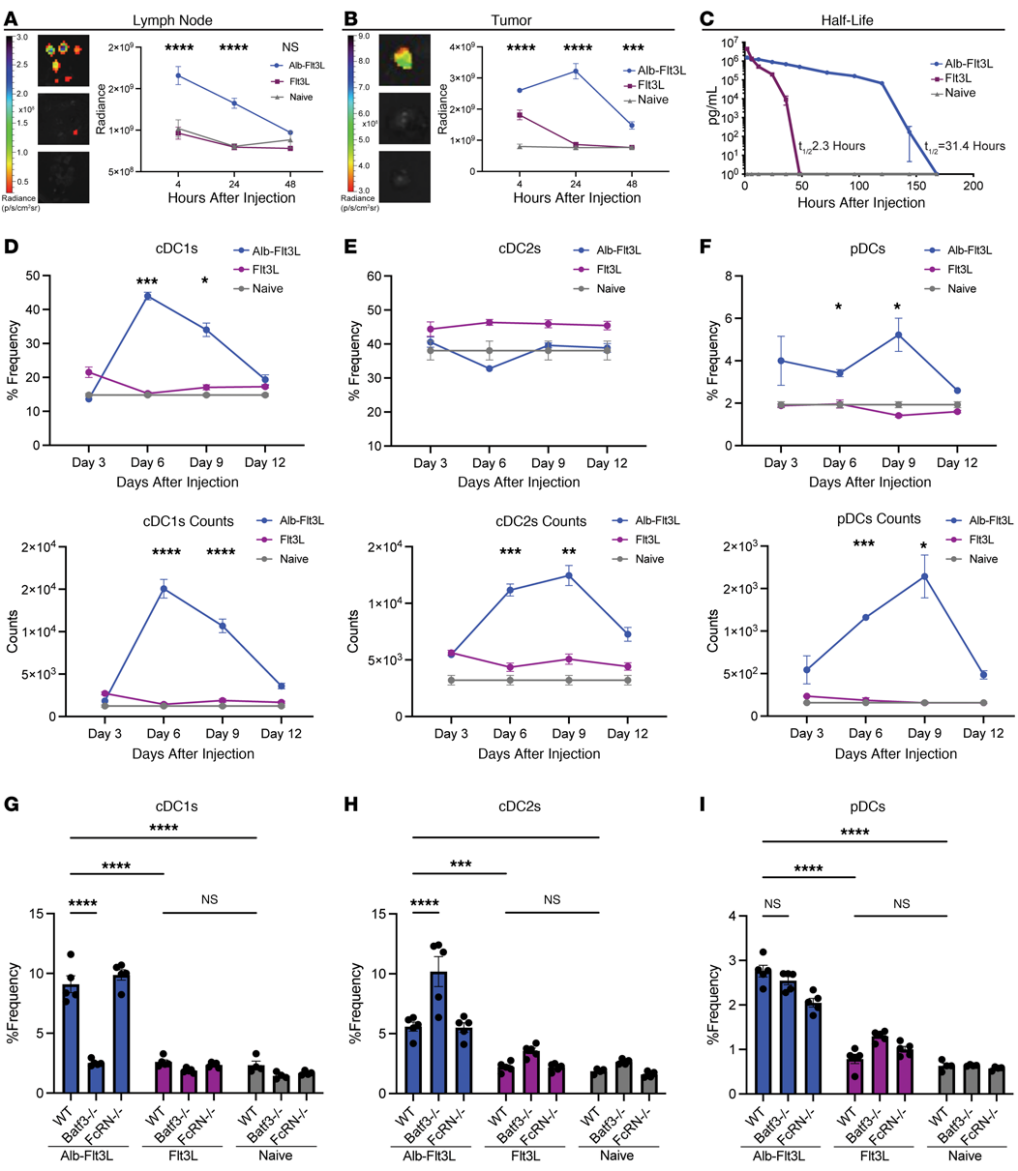
Alb-Flt3L has favorable pharmacokinetic properties and induces superior DC expansion compared with Flt3L. C57BL/6 mice were inoculated with 2 × 10^5^ TC-1 tumor cells and utilized to determine tissue localization and biodistribution once tumors reached approximately 0.5 cm in diameter. Alexa Fluor 647–labeled Alb-Flt3L or Flt3L was injected intravenously at equimolar amounts. Four, 24, and 48 hours after injection, mice were euthanized and tissues were imaged for fluorescent activity using IVIS. (**A**) Representative images of lymph nodes (inguinal, axillary, and brachial) obtained from mice as described and kinetics curves showing average radiance at the indicated time points (*n* = 5). (**B**) Representative images of tumors taken from mice as described and kinetics curves showing average radiance at the indicated time points. In **A** and **B**: top image, Alb-Flt3L; middle image, Flt3L; bottom image, PBS control (*n* = 5). (**C**) Half-life of Alb-Flt3L or Flt3L. Mice were injected with Alb-Flt3L or Flt3L at equimolar amounts. Blood was collected at the indicated time points and serum was separated (*n* = 3). Anti-Flt3L ELISA was performed to determine the amount of Alb-Flt3L or Flt3L remaining in circulation. Curves show detected levels and half-life (*t*_1/2_) calculated by curve fit. To explore kinetics of DC expansion in vivo, C57BL/6 mice were injected with either Flt3L or Alb-Flt3L. Mice were euthanized and spleens were collected on the indicated days. Cells from the spleens were used for flow cytometry. (**D**–**F**) Representative gating, percentage frequency, and counts respectively of (**D**) cDC1s, (**E**) cDC2s, and pDCS (**F**) (*n* = 3). To assay in vivo expansion of DCs in diverse conditions, WT, *FcRn^–/–^*, or *Batf3^–/–^* C57BL/6 mice were injected with Alb-Flt3L, Flt3L, or vehicle control (naive). Five days later, mice were euthanized and splenocytes were analyzed by flow cytometry. (**G**–**I**) Frequency of (**G**) cDC1s, (**H**) cDC2s, or (**I**) pDCs following treatment of the indicated mice with the indicated condition (*n* = 5). Significance determined using 2-way ANOVA. *P < 0.05; **P < 0.01; ***P < 0.001; ****P < 0.0001.

First, we asked whether albumin fusion to Flt3L would allow for Alb-Flt3L to accumulate in the LNs and tumors of mice. Flt3L and Alb-Flt3L were labeled using a succinimidyl ester labeling strategy to covalently attach Alexa Fluor 647. TC-1 tumor–bearing C57BL/6 mice were injected intravenously with 10 μg of Alexa Fluor 647–Flt3L or 50 μg of Alexa Fluor 647–Alb-Flt3L. Mice were euthanized at 4, 24, and 48 hours after injection and tissues were used for fluorescence imaging studies by IVIS. When analyzing fluorescence, Alb-Flt3L displayed significantly higher accumulation in the LNs (Figure 1A) and tumors (Figure 1B) compared with Flt3L- and control-treated groups. Minimal to no signal was observed with Flt3L, suggesting that it does not significantly traffic to the LN or tumor and instead may be circulating in the body until it encounters its receptor and engages. When examining accumulation in other tissues, a significantly higher amount of Alb-Flt3L was observed in the spleen 4 hours after injection, but this signal rapidly decreased to control levels, suggesting that Alb-Flt3L continued circulating to other tissues (Supplemental Figure 1E). As expected, signal was observed for both Flt3L and Alb-Flt3L in the kidneys and liver (Supplemental Figure 1E).

To control for the size difference between Alb-Flt3L and Flt3L and the potential effect this could have on the availability or number of labeling sites, we performed 2 additional control experiments in which we injected a equimolar amounts of either Flt3L or Alb-Flt3L based on protein concentration or equal amounts of fluorescent activity. Tissues were collected from both groups and analyzed by IVIS. For tumors and LNs in mice injected with equal amounts of protein (Supplemental Figure 1, F and H) or equal fluorescence (Supplemental Figure 1, G and I), there was a modest but significantly higher amount of protein detected in the Alb-Flt3L treatment group compared with controls. We also evaluated protein accumulation in the liver, kidney, and spleen of mice injected with either protein at equal concentration (Supplemental Figure 1, J, L and N) or equal fluorescence (Supplemental Figure 1, K, M, and O). We observed similar findings except in the spleen, where a significant difference was not observed between Alb-Flt3L and Flt3L. This is not surprising, given that in our experiment utilizing equimolar amounts of Alb-Flt3L and Flt3L, signal rapidly decreased in the spleen and no significant difference was observed by 24 hours.

In all conditions explored, Alb-Flt3L signal persisted for significantly longer than Flt3L, suggesting that its half-life may be longer. To quantitatively determine the half-life, Flt3L or Alb-Flt3L was injected intravenously into C57BL/6 mice. Blood was collected over 1 week and serum was used in an anti-Flt3L ELISA to determine half-life. Following curve-fit analysis, the half-life of Flt3L in our system was determined to be approximately 2.3 hours, whereas the half-life of Alb-Flt3L was approximately 31.4 hours (Figure 1C), a substantial extension.

As we observed that Alb-Flt3L was present in many tissues throughout the body, we wanted to assess for potential tissue toxicity. Following treatment of C57BL/6 mice with Alb-Flt3L, the heart, lung, liver, pancreas, and kidneys were removed, fixed, and stained with H&E. No significant changes in tissues or inflammatory infiltrates were noted between vehicle- and Alb-Flt3L–treated animals (Supplemental Figure 2A).

### Bioactivity of Alb-Flt3L.

Next, we aimed to determine whether the fusion of albumin with Flt3L affected biological activity, given the markedly different pharmacokinetic profile. Bone marrow progenitor cells were collected from C57BL/6 mice and cultured for 7 days with equimolar amounts of Flt3L or Alb-Flt3L or with GM-CSF as a control. Cells derived from *Batf3^–/–^* and *FcRn*^-/-^ mice were also used. Batf3 is known to be indispensable in cross-presenting-DC biology (43), while FcRn has been shown to be a major factor in the ability of albumin to extend half-life (39, 40). At the end of the culture, cells were collected and used for flow cytometric analysis of DC populations. When analyzing cDC1s (CD11c^+^CD103^+^CD24^+^), Flt3L and Alb-Flt3L both drove substantial but equal expansion of this population (Supplemental Figure 3, B, E, and H). Markedly less cDC1 expansion was observed in the presence of GM-CSF, as expected (Supplemental Figure 3, B, E, and H). Batf3 deficiency restricted cDC1 expansion induced by Flt3L and Alb-Flt3L, confirming that both were signaling through the predicted pathway. cDC2 (CD11c^+^CD11b^+^CD24^–^) populations were observed in the presence of all 3 different cytokines and was not affected by Bat3 deficiency, as expected (Supplemental Figure 3, C, F, and I). Flt3L and Alb-Flt3L were both able to expand pDCs to a greater degree than GM-CSF (Supplemental Figure 3, D, G, and J). Interestingly, Flt3L and Alb-Flt3L both drove substantially greater expansion of pDCs (CD11c^+^SiglecH^+^MHCII^lo^) from *Batf3^–/–^* progenitor cells compared with WT. In all 3 conditions with cells from all 3 mice, FcRn did not affect the expanded populations (Supplemental Figure 3), likely because the role of half-life extension by albumin would be negligible in vitro and would be predicted to have a greater impact in vivo.

### In vivo expansion of cross-presenting DCs following a single treatment with Alb-Flt3L.

Given that Alb-Flt3L was shown to be functional in vitro, we aimed to determine the impact of its improved pharmacokinetic properties in vivo. We wanted to know whether a single injection of Alb-Flt3L would be sufficient to drive expansion of DCs, and what the kinetics of this expansion would be. Flt3L, Alb-Flt3L, or vehicle control (naive) was injected intravenously into WT mice. Then, every 3 days for 12 days in total, mice were euthanized, and DC expansion was measured following gating and lineage exclusion on CD3, CD19, NK1.1, and GR-1 (Supplemental Figure 4). For cDC1s (live Lin^–^CD11c^+^XCR1^+^MHCII^+^), Alb-Flt3L induced superior expansion compared with Flt3L, with the peak of expansion happening around day 6 (Figure 1D and Supplemental Figure 5A). For cDC2s (live Lin^–^CD11c^+^CD11b^+^SIRPa^+^), similar frequencies were seen over time, but significant expansion was seen with Alb-Flt3L according to total counts (Figure 1E and Supplemental Figure 5B). Finally, we observed that Alb-Flt3L also significantly expanded pDCs (live Lin^–^CD11c^+^SiglecH^+^B220^+^) (Figure 1F and Supplemental Figure 5C), although it did seem that the peak of expansion for pDCs was somewhat later, around day 9. Together, these data support the notion that Alb-Flt3L significantly expands DC subsets, particularly cDC1s and pDCs following a single injection.

Next, we explored the role of Batf3 and FcRn in the expansion of DCs in vivo following Alb-Flt3L administration, as in our in vitro study. WT, *Batf3^–/–^*, or *FcRn^–/–^* mice were administered equimolar amounts of Alb-Flt3L or Flt3L. Five days later, mice were euthanized and DC expansion was assayed. Alb-Flt3L drove significantly greater expansion of cDC1s as compared with Flt3L (Figure 1G and Supplemental Figure 5D), likely due to its enhanced pharmacokinetics properties. Importantly, this expansion of cDC1s by Alb-Flt3L was not observed in *Batf3^–/–^* mice, confirming that Alb-Flt3L is expanding cDC1s in a Batf3-dependent manner (Figure 1G and Supplemental Figure 5D). FcRn deficiency did not affect the in vivo expansion of DCs induced by Alb-Flt3L (Figure 1, G–I, and Supplemental Figure 5, D–F). Alb-Flt3L also significantly expanded cDC2s and pDCs compared with Flt3L (Figure 1, H and I, and Supplemental Figure 5, E and F). When analyzing pDC expansion in *Batf3^–/–^* mice, we observed significant expansion of pDCs just as in our in vitro experiment (Figure 1I and Supplemental Figure 5F).

In addition to our flow cytometric studies, we wanted to ensure that Alb-Flt3L was not having adverse effects in the blood. Five days following treatment as indicated, blood was collected and used for a complete blood count, revealing no major changes except in monocytes, as expected with Alb-Flt3L treatment (Supplemental Figure 6). We also checked for the presence of deleterious antibodies against albumin, Flt3L, or Alb-Flt3L following 3 weekly injections. Serum was collected from the mice 1 month after the initial injection and tested by ELISA. Anti-Flt3L or anti-albumin commercial antibodies were used as positive controls. No deleterious antibodies were detected (Supplemental Figure 6, B–D). Altogether, these results support the idea that Alb-Flt3L is able to induce superior DC expansion, particularly of cross-presenting cDC1s compared with Flt3L, and is therefore likely better suited to be a therapeutic agent.

### Alb-Flt3L monotherapy leads to intratumoral expansion of DCs, T cell activation, and tumor control.

**Figure 2:**
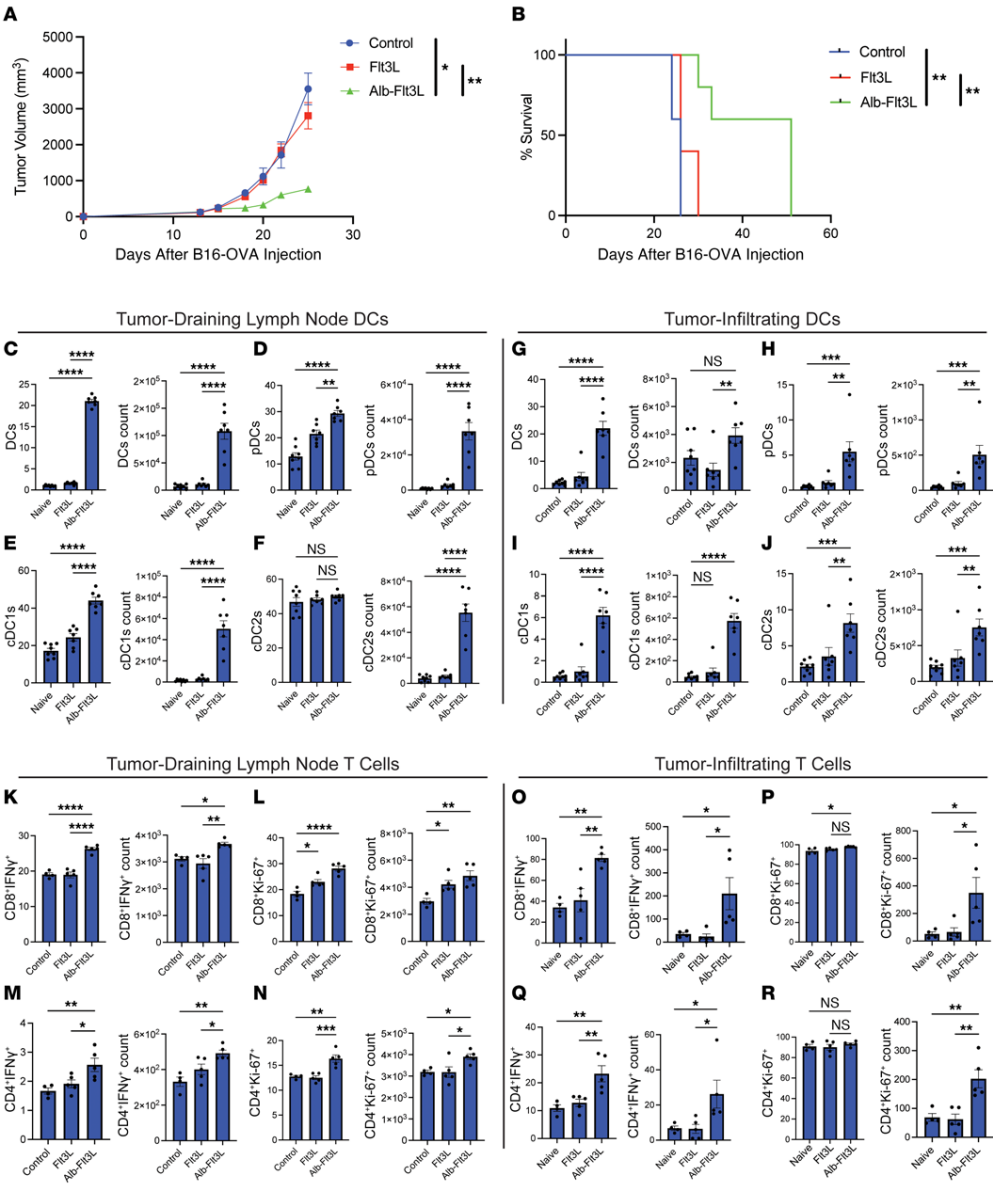
Treatment with Alb-Flt3L drives cross-presenting-DC expansion, T cell activation, and ultimately tumor control. C57BL/6 mice were inoculated with 2 × 10^5^ B16-OVA tumor cells subcutaneously. Sixteen days after implantation, mice were administered Alb-Flt3L (100 μg), Flt3L (20 μg) (equimolar amounts), or vehicle control on days 19, 21, 24, and 30 for a total of 4 doses. (**A**) Tumor growth and (**B**) survival curves following the described treatment protocol (*n* = 5). For immunologic analysis, the experiment was repeated, and mice were sacrificed following 3 treatments (day 24) with Alb-Flt3L, Flt3L (equimolar amounts), or vehicle control, and assayed for DC expansion and T cell activation. The frequencies and counts of (**C**) total DCs (**D**) pDCs, (**E**) cDC1s, and (**F**) cDC2s in the tumor-draining lymph nodes and the frequencies and counts of (**G**) total DCs, (**H**) pDCs, (**I**) cDC1s, and (**J**) cCD2s in the tumor for the indicated treatment groups are shown (*n* = 7). The frequencies and counts of (**K**) CD8^+^IFN-γ^+^, (**L**) CD8^+^Ki-67^+^, (**M**) CD4^+^IFN-γ^+^, and (**N**) CD4^+^Ki-67^+^ cells in the tumor-draining lymph nodes and the frequencies and counts of (**O**) CD8^+^IFN-γ^+^, (**P**) CD8^+^Ki-67^+^, (**Q**) CD4^+^IFN-γ^+^, and (**R**) CD4^+^Ki-67^+^ cells in the tumors for the indicated treatment groups are shown (*n* = 5). *P < 0.05; **P < 0.01; ***P < 0.001; ****P < 0.0001 by 1-way ANOVA (bar graphs) or 2-way ANOVA (tumor growth curves), except tumor survival curves in **B**, which were assessed using the log-rank test.

Given our results showing that Alb-Flt3L has favorable trafficking properties, half-life, and ability to expand DCs in vivo compared with Flt3L, we wanted to explore its therapeutic utility in cancer. B16-OVA tumor cells, a model of melanoma expressing the chicken ovalbumin (OVA) antigen, was injected into C57BL/6 mice. Once the tumors reached 200 mm^3^ in volume, approximately 16 days after implantation, mice were treated with either 100 μg of Alb-Flt3L or 20 μg of Flt3L intravenously (equimolar ratio). Additional treatments were administered on days 19, 21, 24, and 30, given that these were aggressive, established tumors. We observed significant tumor control and enhanced survival (Figure 2, A and B) in mice treated with Alb-Flt3L compared with Flt3L or vehicle control. Sixty percent of mice became tumor free and survived long term, an impressive therapeutic achievement for an aggressive tumor system receiving a monotherapy.

Next, we wanted to explore the immunological factors that could be promoting this improved tumor control. We repeated this experiment, but instead sacrificed mice after 3 treatments with Alb-Flt3L or Flt3L, as we believed that his would allow enough time for the immunological factors at play to become clear. First, we assayed DCs in the tumor-draining LNs. By both frequency and count, we observed significant expansion of total DCs (Figure 2C), pDCs (Figure 2D), and cDC1s (Figure 2E) in mice treated with Alb-Flt3L. cDC2s were significantly expanded in number, but not frequency, in Alb-Flt3L–treated mice. Similarly, we observed significant expansion by frequency and count of total DCs (Figure 2G), pDCs (Figure 2H), and cDC1s (Figure 2I) in the tumors of mice treated with Alb-Flt3L. The same pattern was observed for cDC2s (Figure 2J).

With expanded DCs in the tumor-draining LNs and tumor following Alb-Flt3L treatment, presumably carrying antigens from the B16-OVA tumor inoculated in these mice, we hypothesized that this would lead to the activation and proliferation of T cells in these tissues. There was a significantly higher number and frequency of CD8^+^ T cells (Figure 2, K and L) and CD4^+^ T cells (Figure 2, M and N) expressing IFN-γ and Ki-67 in the tumor-draining LNs. When examining the tumor, there was a significantly higher number and frequency of IFN-γ–positive CD8^+^ (Figure 2O) and CD4^+^ (Figure 2Q) T cells alongside a significantly higher number, but not frequency of CD8^+^ (Figure 2P) and CD4^+^ (Figure 2R) T cells expressing Ki-67. The gating strategies used for DCs and T cells in these experiments are included in Supplemental Figures 7 and 8. Altogether, these results support the notion that Alb-Flt3L, likely through its tissue accumulation and long half-life, can accumulate in the tumor-draining LNs and tumor, where it can expand cross-presenting DCs and allow for substantially improved priming and activation of T cells, ultimately leading to tumor control and long-term survival.

### In situ vaccination with Alb-Flt3L plus radiation therapy leads to tumor control and neoantigen-specific immunity.

**Figure 3:**
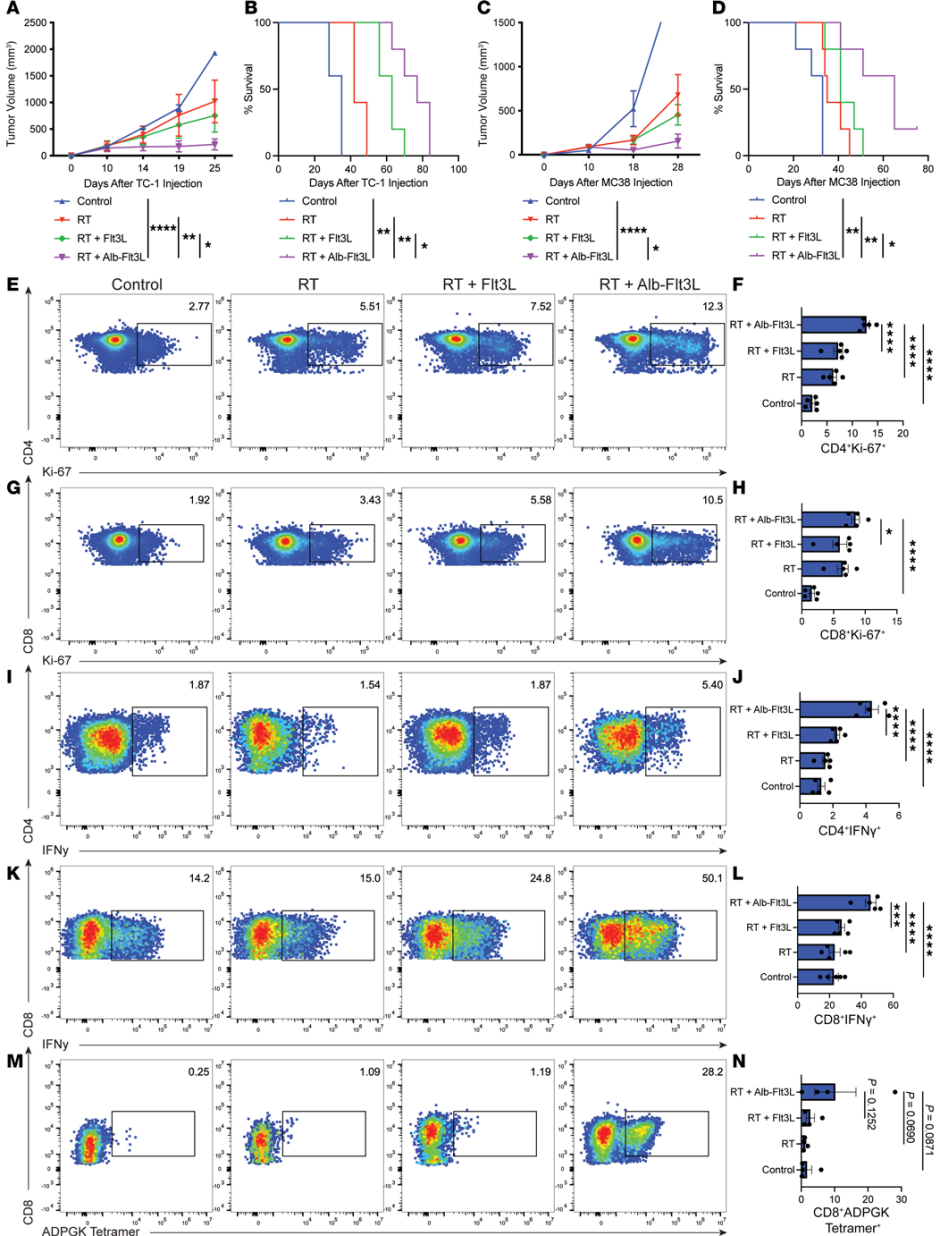
Alb-Flt3L plus radiation therapy leads to superior tumor control and induction of spontaneous neoantigen-specific CD8^+^ T cells. C57BL/6 mice were inoculated with 2 × 10^5^ TC-1 tumor cells subcutaneously. Ten days later, following establishment of tumors, mice were administered Flt3L, Alb-Flt3L (equimolar amounts), or vehicle control intravenously. Twenty-four hours later, mice were treated with 10 Gy of CBCT targeted radiation therapy (RT) using the SARRP. Mice were treated with Flt3L, Alb-Flt3L, or vehicle control every 5 days for a total of 4 doses. (**A**) Tumor growth and (**B**) survival curves following the described treatment protocol (*n* = 5). Similar to Figure 2, A and B, but instead C57BL/6 mice were inoculated with 4 × 10^5^ MC38 tumor cells subcutaneously. Mice were treated identically and monitored to determine (**C**) tumor growth and (**D**) survival curves (*n* = 5). Immune responses observed in mice treated with Alb-Flt3L + RT. PBMCs collected 2 weeks after initiation of treatment were assessed for activation marker expression. Representative flow gating and quantification of (**E** and **F**) CD4^+^Ki-67^+^, (**G** and **H**) CD8^+^Ki-67^+^, (**I** and **J**) CD4^+^IFN-γ^+^, and (**K** and **L**) CD8^+^IFN-γ^+^ cells for the indicated treatment groups (*n* = 5). (**M** and **N**) For the assessment of MC38 neoantigen–specific CD8^+^ T cells in mice treated with Alb-Flt3L + RT, tumors were collected and single-cell suspensions prepared. Cells were stained with lineage markers and CD8^+^ADPGK-tetramer^+^ cells were gated as shown in the representative flow gating histograms (**M**) and summary data are shown (*n* = 4) (**N**). All figures display the percentage frequency of CD8^+^ or CD4^+^ T cells (percentage of parental cells). *P < 0.05; **P < 0.01; ***P < 0.001; ****P < 0.0001 by way ANOVA (bar graphs) or 2-way ANOVA (tumor growth curves), except tumor survival curves in **B** and **D**, which were assessed using the log-rank test.

Having observed the ability of Alb-Flt3L to expand DCs in vivo, we wanted to explore its ability to serve as a cancer therapeutic agent alongside radiation therapy, which is standard of care for many diseases. TC-1 tumors, a model of high-risk HPV-associated cancer, were implanted in C57BL/6 mice 10 days before treatment began. Mice were administered Alb-Flt3L or Flt3L in equimolar amounts followed by 10 Gy of targeted radiation therapy. The logic of this experimental design was that Alb-Flt3L would promote the expansion of cross-presenting DCs in the tumors and draining LNs. Radiation therapy would allow for antigen release from the tumor, which could then be taken up by local cDC1s, processed, and cross-presented on MHCI to tumor-specific cytotoxic T cells. Alb-Flt3L plus radiation treatment resulted in significantly better tumor control (Figure 3A) and overall survival (Figure 3B) compared with Flt3L plus radiation, radiation alone, or untreated controls. We tested this hypothesis in a second tumor system, MC38, a model of colon adenocarcinoma. In MC38, we also observed superior tumor control (Figure 3C) and overall survival (Figure 3D) in mice treated with Alb-Flt3L. We explored the mechanism that was driving this phenomenon. PBMCs were prepared from MC38 tumor–bearing mice treated prepared as described above and were used for immune cell analysis by flow cytometry. We observed a significantly higher percentage of CD4^+^Ki-67^+^ (Figure 3, E and F) and CD8^+^Ki-67^+^ (Figure 3, G and H) cells in mice treated with Alb-Flt3L plus radiation, suggesting that our treatment regimen was likely driving T cell activation and subsequent proliferation of both CD4^+^ and CD8^+^ T cells. We also observed robust IFN-γ production by both CD4^+^ (Figure 3, I and J) and CD8^+^ (Figure 3, K and L) T cells in mice treated with Alb-Flt3L plus radiation. This supports the idea that treatment with Alb-Flt3L plus radiation not only drives activation, but also greater effector function of T cells, which likely contributes to our observed antitumor effects.

Given that we observed marked tumor control and T cell activation in mice treated with Alb-Flt3L plus radiation, we sought to determine the antigen specificity of these T cells. We hypothesized that these activated T cells are likely recognizing neoantigens expressed in MC38 tumors that were likely released in large quantities following the administration of radiation. Previous reports have detailed the mutational profile and neoantigens expressed by MC38 tumors (44). We selected one of these neoantigens that was demonstrated to have high immunogenicity in vaccination studies, ADPGK, and acquired an ADPGK tetramer. We collected tumor-infiltrating T cells from these mice and assessed whether any of the CD8^+^ T cells in these tumors were specific for ADPGK neoantigen. We found a large population of CD8^+^ T cells in mice treated with Alb-Flt3L plus radiation that were specific for ADPGK (Figure 3, M and N), supporting the idea that Alb-Flt3L potentially serves as an in situ vaccination strategy to uncover antigens and mount tumor-specific cytotoxic T cell immunity regardless of the antigen profile in an individual tumor. It is important to note however, that this ADPGK neoantigen–specific response was not seen in all mice, and the finding itself is not statistically significant (Figure 3, M and N). This makes sense, as in the absence of vaccination against a specific neoantigen or strong immunodominance of one antigen, it would be difficult to predict exactly which epitope would be dominant in a genetically identical mouse with a unique T cell repertoire.

We were curious whether these ADPGK neoantigen–specific T cells found in mice treated with Alb-Flt3L plus radiation were clonally expanded from a single cell, or consisted of a diverse pool of unique T cell receptors (TCRs) with shared antigen specificity. To address this, we sorted CD8^+^ ADPGK-tetramer^+^ cells and performed TCRβ chain sequencing. This revealed a wide array of unique rearrangements in each mouse assayed, suggesting that many different T cells were being activated by ADPGK-loaded cDC1s and infiltrating the tumor (Supplemental Figure 9). We also assessed the profile of all tumor-infiltrating CD8^+^ T cells, revealing greater clonality in mice treated with Alb-Flt3L plus radiation (Supplemental Figure 9). Together, these results support the notion that Alb-Flt3L can drive cDC1 expansion, antigen uptake and loading, and subsequent tumor neoantigen–specific T cell activation and tumor control spontaneously.

### Alb-Flt3L plus cisplatin chemotherapy treatment leads to tumor control and improved survival.

**Figure 4:**
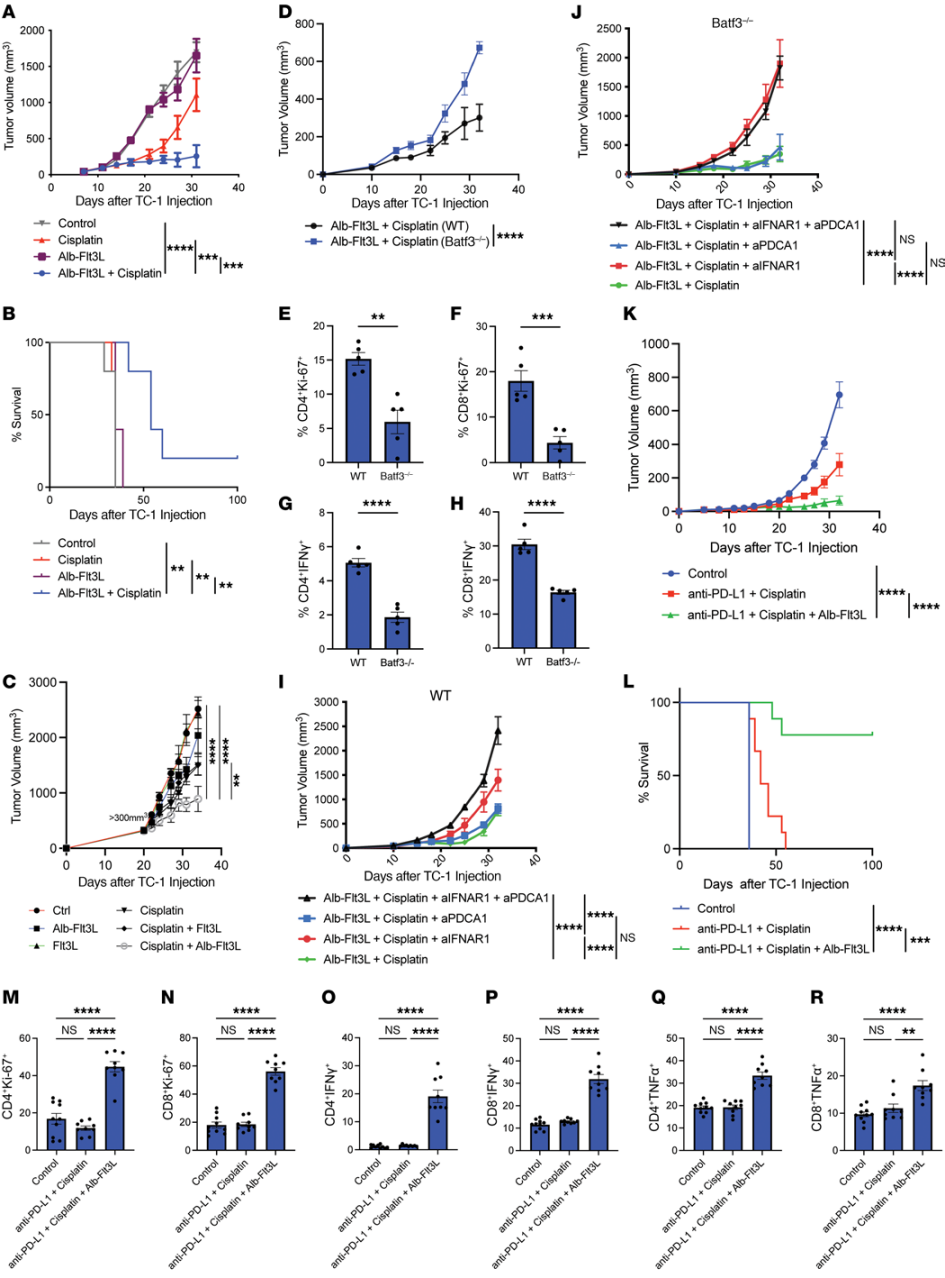
Alb-Flt3L synergizes with chemotherapy and anti–PD-L1 to promote superior Batf3-dependent tumor control. C57BL/6 mice were inoculated with 2 × 10^5^ TC-1 tumor cells subcutaneously. Eight days later, mice were treated with Alb-Flt3L followed by cisplatin 24 hours later. The treatment regimen consisting of Alb-Flt3L followed by cisplatin was performed every 5 days for a total of 3 times. (**A**) Tumor growth and (**B**) survival curves of TC-1 tumor–bearing mice treated with the indicated reagents (*n* = 5). For the assessment of tumor control in large tumors, C57BL/6 mice were inoculated with 2 × 10^5^ TC-1 tumor cells subcutaneously (*n* = 5). Tumors were allowed to grow until they reached more than 300 mm^3^, which was approximately 21 days. Mice were then treated with 100 μg of Alb-Flt3L or 20 μg of Flt3L (equimolar amounts); cisplatin was administered 24 hours later. This regimen was administered approximately every 3 to 4 days for a total of 3 cycles. Alb-Flt3L or Flt3L treatment continued every approximately 3 days. (**C**) Tumor growth curves for TC-1 large tumor experiment. WT C57BL/6 mice were inoculated with 2 × 10^5^ TC-1 tumor cells (*n* = 5). Twenty days later, the mice were treated with Alb-Flt3L, Flt3L, cisplatin, cisplatin + Flt3L, cisplatin + Alb-Flt3L, or vehicle (control). (**D**) Tumor growth curves following treatment of WT or *Batf3^–/–^* mice with Alb-Flt3L + cisplatin (*n* = 5). PBMCs were collected from mice 20 days after initiation of treatment and prepared for flow cytometry. Percentages of (**E**) CD4^+^Ki-67^+^, (**F**) CD8^+^Ki-67^+^, (**G**) CD4^+^IFN-γ^+^, and (**H**) CD8^+^IFN-γ^+^ cells in WT or *Batf3^–/–^* mice treated with Alb-Flt3L + cisplatin (*n* = 5). Data shown are the percentage of parental cells (CD4^+^ or CD8^+^ T cells) (*n* = 5). TC-1–bearing WT C57BL/6 mice were administered blocking or depleting antibodies as indicated and treated with Alb-Flt3L + cisplatin and (**I**) tumor growth was determined (*n* = 5). (**J**) Similar to **I**, but in *Batf3^–/–^* mice (*n* = 5). TC-1 tumor–bearing mice were treated with 4 doses of anti–PD-L1 every 3 days starting on day 5. In addition, Alb-Flt3L was administered every 3 days starting on day 5. Cisplatin was administered on days 21 and 28 after tumor inoculation. (**K**) Tumor growth and (**L**) survival curves for the indicated treatment groups (*n* = 9). PBMCs were collected 30 days following tumor inoculation and prepared for flow cytometry. Frequency of (**M**) CD4^+^Ki-67^+^, (**N**) CD8^+^Ki-67^+^, (**O**) CD4^+^IFN-γ^+^, (**P**) CD8^+^IFN-γ^+^, (**Q**) CD4^+^TNF-α^+^, and (**R**) CD8^+^TNF-α^+^ cells among PBMCs in the indicated treatment groups. Data shown are the percentages of parental cells (CD4^+^ or CD8^+^ T cells) (*n* = 9). **P < 0.01; ***P < 0.001; ****P < 0.0001 by 1-way ANOVA (bar graphs) or 2-way ANOVA (tumor growth curves), except tumor survival curves in **B** and **L**, which were assessed using the log-rank test, and 2-group comparisons, which used the 2-tailed Student’s *t* test (**E**–**H**).

Having observed significant tumor control by Alb-Flt3L plus radiation, we sought to determine whether the same findings could be achieved with chemotherapy administered as cisplatin in place of radiation therapy. C57BL/6 mice were injected with TC-1 tumors, and the mice were administered Alb-Flt3L followed by cisplatin chemotherapy as in our radiation experiment. Mice were treated throughout the experimental period. We observed significantly improved tumor control (Figure 4A) and overall survival (Figure 4B) in mice treated with Alb-Flt3L plus cisplatin compared with Alb-Flt3L alone, cisplatin alone, or untreated controls. This result enhances the translational versatility of Alb-Flt3L as an in situ vaccination strategy.

### Control of large established tumors using Alb-Flt3L as a monotherapy or in combination with cisplatin.

Many groups believe that the success of a therapeutic regimen in palpable, but not fully established, murine tumors is not predictive of clinical success in human cancer. Accordingly, we sought to determine whether Alb-Flt3L could mediate tumor control in large established murine tumors. First, we tested whether Alb-Flt3L plus cisplatin treatment could still lead to tumor control if treatment was considerably delayed. TC-1 tumors were injected into C57BL/6 mice, and we waited until the tumor volume in each mouse was over 300 mm^3^, approximately 20 days. We then began our intravenous treatment of Alb-Flt3L or Flt3L at a higher dose of 100 μg or 20 μg, respectively, followed by cisplatin. Treatment continued and tumor size was monitored. We observed the best tumor control in mice treated with Alb-Flt3L plus cisplatin, in which tumor volume was significantly smaller than all control arms, including Flt3L plus cisplatin (Figure 4C). These data suggest that Alb-Flt3L may have clinical promise for the treatment of established or advanced cancers.

### Mechanism of tumor control with Alb-Flt3L plus cisplatin treatment.

Given the role of Batf3 in Alb-Flt3L–mediated cross-presenting-DC expansion, we wanted to determine whether this pathway could also be implicated in Alb-Flt3L–mediated tumor control. WT or *Batf3^–/–^* mice were implanted with TC-1 tumors and treated with Alb-Flt3L plus cisplatin, as in our previous experiments. Tumor size was monitored, revealing that *Batf3^–/–^* mice treated with Alb-Flt3L plus cisplatin failed to control tumors, which grew significantly faster than those of WT controls (Figure 4D). We also explored immunologic factors that could be contributing. This revealed that *Batf3^–/–^* mice failed to induce large populations of proliferating CD4^+^ (Figure 4E) or CD8^+^ (Figure 4F) T cells determined by Ki-67 staining in flow cytometry. Effector function of T cells was also affected, as significantly less IFN-γ production was observed by CD4^+^ (Figure 4G) and CD8^+^ (Figure 4H) T cells in *Batf3^–/–^* mice. This confirms that Alb-Flt3L, as with Flt3L, requires Batf3 in order to successfully expand cDC1s. Without Batf3, these DCs cannot develop, which limits downstream T cell activation, and ultimately ablates tumor control. It should be noted that depressed T cell activity is seen to some degree in *Batf3^–/–^* mice, but the model still permits investigation of DC-dependent T cell immunity.

In Figure 1 where we explored all the DC populations that were expanded by Alb-Flt3L treatment, we observed significant differentiation of progenitor cells into pDCs, a cell population that is generally not believed to be developmentally Batf3 dependent (45). Given that pDCs are known to be one of the major sources of type I IFNs (TI-IFNs) (46), and cytotoxic therapies such as chemotherapy and radiation therapy are known to cause large amounts of TI-IFN release (47, 48), we wanted to explore the contribution of pDCs and TI-IFNs in our observed tumor control following treatment with Alb-Flt3L plus cisplatin. C57BL/6 mice were injected with TC-1 tumors and treated with Alb-Flt3L plus cisplatin, as in our other experiments. In addition, mice were administered either anti-IFNAR1 to block TI-IFN signaling, anti–PDCA-1 to deplete pDCs, or with both anti-IFNAR1 and anti–PDCA-1. While pDC depletion alone did not have a big impact on tumor growth, IFNAR1 blockade did have an effect and combination pDC depletion and IFNAR1 blockade had the greatest impact (Figure 4I). We also explored these pathways in the absence of Batf3, revealing that IFNAR1 blockade and combination pDC depletion and IFNAR1 blockade had similar effects (Figure 4J). Together, these results suggest that Batf3, TI-IFN signaling, and pDCs are all likely important in Alb-Flt3L–mediated tumor control to varying degrees.

### Alb-Flt3L plus cisplatin strongly synergizes with immune checkpoint blockade.

Given the widespread adaptation and clinical promise of immune checkpoint blockade strategies, we wanted to explore potential synergy with Alb-Flt3L. Hypothetically, checkpoint blockade would “release the brakes” from T cells and sensitize them to activation by tumor antigen–loaded cross-presenting DCs that were expanded by Alb-Flt3L. To test this hypothesis, TC-1 tumors were implanted in C57BL/6 mice. Mice were treated with anti–PD-L1 intraperitoneally and Alb-Flt3L intravenously, followed by cisplatin intraperitoneally. We observed significantly better tumor control (Figure 4K) and overall survival (Figure 4L) with anti–PD-L1 plus cisplatin and Alb-Flt3L treatment compared with control arms. Long term, 70% of mice were clear of any tumor. This is a substantial improvement compared with Alb-Flt3L plus cisplatin therapy in Figure 4B where only 20% of mice survived long term. Immunological analysis provided context for this result, as significantly higher percentages of proliferating CD4^+^Ki-67^+^ (Figure 4M) and CD8^+^Ki-67^+^ (Figure 4N) T cells were observed in the combination treatment condition. T cell effector function, assayed as CD4^+^IFN-γ^+^ (Figure 4O), CD8^+^IFN-γ^+^ (Figure 4P), CD4^+^TNF-α^+^ (Figure 4Q), and CD8^+^TNF-α^+^ (Figure 4R) cells, was also significantly higher in animals treated with anti–PD-L1 plus cisplatin and Alb-Flt3L.

### Human cross-presenting-DC expansion by Alb-Flt3L in CD34^+^ stem cell–humanized mice.

**Figure 5:**
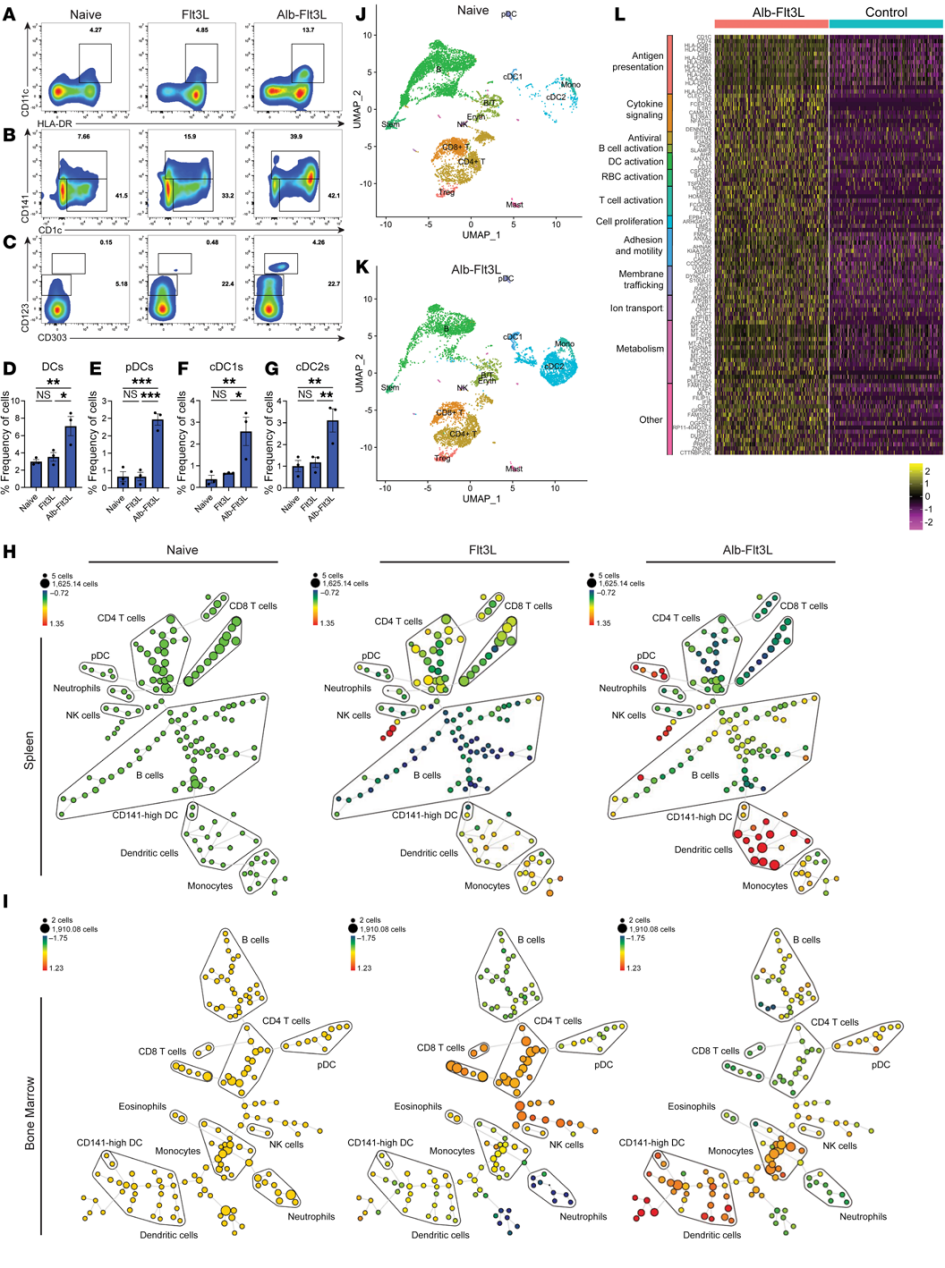
Expansion of human DC populations by Alb-Flt3L in humanized mice. NSG mice were irradiated and engrafted with human CD34^+^ cells according to Taconic’s established protocols. Successful chimerism was confirmed by flow cytometry. Three mice from each donor were generated, allowing for a donor-match control to be in each group (*n* = 3). Mice were administered Flt3L, Alb-Flt3L (equimolar amounts), or vehicle control every 5 days for a total of 3 doses. Mice were then euthanized, and spleens and bone marrow were harvested and used for immune cell analysis. Representative flow gating and percentages of total DCs gated as CD11c^+^HLA-DR^+^ (**A** and **D**), cDC1s gated as CD11c^+^HLA-DR^+^CD141^+^ (**B** and **F**), cDC2s gated as CD11c^+^HLA-DR^+^CD1c^+^ (**B** and **G**), and pDCs gated as CD11c^+^HLA-DR^lo^CD123^+^CD303^+^ (**C** and **E**). Representative SPADE analysis of flow cytometry data collected from (**H**) spleen or (**I**) bone marrow of mice treated with vehicle, Flt3L, or Alb-Flt3L (equimolar amount). Donor-matched controls are shown. Vehicle was used to set baseline cell populations in each cluster. scRNA-seq data collected from donor-matched spleens of mice treated with vehicle or Alb-Flt3L. UMAP projection of scRNA-seq data from (**J**) vehicle-treated (naive) or (**K**) Alb-Flt3L–treated mice. Mono, monocytes. (**L**) Heatmap showing gene cluster differences in mice treated with Alb-Flt3L compared with control. *P < 0.05; **P < 0.01; ***P < 0.001 by 2-tailed Student’s *t* test.

Finally, as the goal of development for Alb-Flt3L is clinical translation in the near future, it is essential to determine whether Alb-Flt3L is capable of expanding human DCs, as they are not phenotypically identical to mouse DCs. To test this ability, we utilized CD34^+^ hematopoietic stem cell–humanized NSG mice. Three humanized mice were prepared from the same human CD34^+^ cell donor (Supplemental Figure 10, A–C), so that a matched control could be in each treatment arm, including Alb-Flt3L, Flt3L, or vehicle control (naive). Our human albumin–human Flt3L construct was utilized for these studies. Following administration of our treatments, mice were euthanized, spleens and bone marrow were collected, and analyses was performed. Extensive gating was performed to ensure that we were analyzing human DCs and eliminating any residual mouse DCs from our analysis (Supplemental Figure 11A). Multiparameter (19 marker) flow cytometry revealed that Alb-Flt3L drove significant expansion of human DCs versus Flt3L controls, including total DCs (Figure 5, A and D, and Supplemental Figure 11H), cDC1s (Figure 5, B and E, and Supplemental Figure 11I), cDC2s (Figure 5, B and F, and Supplemental Figure 11J), and pDCs (Figure 5, C and G, and Supplemental Figure 11K). The lineages of our cDC1s and cDC2s were confirmed by qPCR analysis on sorted CD1c^+^ or CD141^+^ cells using known transcription factors (Supplemental Figure 11, B–G). We also explored expansion of other immune cell populations and represented these changes in SPADE plots. Naive mice were set as baseline cell populations, and population changes with Flt3L or Alb-Flt3L treatment were investigated. Other than DCs, which we determined were expanded, B cell populations, NK cells, and some monocyte lineage cells were also expanded with Alb-Flt3L treatment in the spleen (Figure 5H) and bone marrow (Figure 5I) of humanized mice. To further characterize changes to human immune cell populations following treatment with Alb-Flt3L, we performed single-cell RNA-seq (scRNA-seq) on sorted human cells from our humanized mice. Alb-Flt3L treatment verses control was analyzed without an Flt3L control, as we believe this would allow us to gauge the types of changes this immunotherapeutic could potentially have in an individual with or without treatment. This confirmed that populations of DCs were expanded following treatment with Alb-Flt3L (Figure 5, J and K). In addition, we observed an Alb-Flt3L–mediated upregulation of genes involved in antigen presentation, cytokine signaling, proliferation, metabolism, and many other pathways from our sequence analysis in all human cells in the humanized mice (Figure 5L). Together, these results confirm that Alb-Flt3L can potently expand human cross-presenting DCs and may serve as a future therapeutic modality for human applications aimed at expanding DC populations.

## Discussion

In our study, we detailed the development of what we believe to be a novel Flt3L-based therapeutic molecule, Alb-Flt3L, that has enhanced pharmacokinetic properties, including LN and tumor targeting and enhanced half-life compared with Flt3L. We demonstrate that Alb-Flt3L induced greater expansion of DCs compared with Flt3L following a single injection. Alb-Flt3L was employed as an in situ vaccination strategy to mount tumor neoantigen–specific immunity without the need for a tumor-specific vaccination. We believe this is because Alb-Flt3L–expanded DCs can take up tumor antigen in the tumor microenvironment that is released by radiation or chemotherapy, process and cross-present these antigens, and expand preexisting pools of neoantigen-specific T cells in the mice, which then mediate tumor control. Alb-Flt3L’s ability to expand DCs was shown to be dependent on Batf3, and Alb-Flt3L’s immunostimulatory ability was shown to depend on TI-IFN signaling and pDCs. Finally, the ability of human Alb-Flt3L to expand human DCs was demonstrated using 19-color flow cytometry and scRNA-seq in CD34^+^ stem cell–humanized mice.

It is important to note that while the trends observed regarding in vivo expansion of cDC1s, cDC2s, and pDCs following administration of Flt3L or Alb-Flt3L were identical throughout our experiments, there were experiment-to-experiment magnitude differences. We believe that this is likely due to slight differences between batches of our Alb-Flt3L and Flt3L preparations, as well as potential differences following flow staining on different days, which would be expected within reason.

When exploring the factors contributing to the enhanced in vivo functionality of Alb-Flt3L, we believed that FcRn would be indispensable, but this was not the case in terms of DC expansion. We did not explore the role of this receptor in our half-life studies, but we believe that there would not be a large impact since a single administration of Alb-Flt3L was still able to promote DC expansion in *FcRn^–/–^* mice (see Figure 1, G–I). There are many potential explanations for this. For one, there are many other putative receptors known to bind albumin, as well as the cubilin-megalin complex, which is known to be important for albumin retrieval in the renal tract (49). These receptors are currently poorly understood and serve as a place for future investigation, particularly for groups developing albumin-containing therapeutics for human use. Several different peptides and small proteins have been fused to albumin to prolong the half-life and/or biological activities (for review, see 50–53). In addition to receptor-mediated recycling, it is also reasonable to think that Alb-Flt3L would have a much longer half-life simply due to its large size compared with Flt3L (54).

Before clinical translation of Alb-Flt3L or additional preclinical studies, a dose escalation and kinetic study would be appropriate. The specific goal would be to determine the amount of Alb-Flt3L that results in the greatest expansion of DCs, and for how long these DCs persist. The effect of Alb-Flt3L on other immune cell populations with differing dosing and kinetics would also be explored in this experiment. These studies would need to be performed in an appropriate model system, as the half-life of human albumin differs from that of mouse albumin. In addition, we could explore these parameters in FcRn-deficient models and human-FcRn-transgenic systems to better elucidate contributing factors. This would best inform clinical usage and dosing of Alb-Flt3L, and we plan to perform this in future studies.

In our MC38 tumor experiments where spontaneous neoantigen-specific T cell immunity was observed following treatment with Alb-Flt3L plus radiation, there was a large range of tetramer^+^ cell numbers within the Alb-Flt3L plus radiation treatment group, but not large amounts in the controls. The large range can likely be explained by the fact that the ADPGK neoantigen is just one of many mutations known to be present in MC38 cells. There have also been virus-associated antigens shown to be immunogenic in these tumors that could be playing a crucial role (55, 56). It would not be expected that each individual mouse would generate large percentages of cells recognizing the same neoantigen, given the diverse profile of potential targets and the independent development of the TCR repertoire in each animal. In future studies, we can investigate the ability of Alb-Flt3L in situ vaccination to generate immunity against a multitude of known antigens expressed by a tumor under different conditions empirically. In addition, the functional profile of these cells can be explored transcriptomically given their limited numbers.

The utility of Alb-Flt3L is most exciting when considering human clinical translation. Flt3L has been under development as a therapeutic for decades. Recently, clinical studies have yielded very exciting results using Flt3L alongside checkpoint blockade and radiotherapy (57). In principle, Alb-Flt3L could be a “plug-and-play” for future trials investigating in situ vaccination with Flt3L. The tissue-targeting ability of Alb-Flt3L would eliminate the need to perform intratumoral injections, a process that can be cumbersome for large-scale studies in a variety of clinical practice environments. In addition, the significantly improved half-life of Alb-Flt3L would substantially lengthen the time needed between injections, and subsequently reduce cost and inconvenience to patients. It is also worth considering that the half-life of human albumin is significantly longer compared with mouse albumin, so the dosing required in human Alb-Flt3L would theoretically be even more infrequent that what we use in preclinical studies (38).

For clinical translation, it will be important to characterize the potential toxicity related to Alb-Flt3L, such as autoimmunity. In the current study, we did not observe meaningful changes in all the vital organs receiving Alb-Flt3L compared with the control group (see Supplemental Figure 2). It will be important to further characterize whether the higher dose of Alb-Flt3L will lead to any toxicity, either histologically or biochemically. We envision that a pilot study should be performed in a limited number of healthy volunteers to determine the dose that Alb-Flt3L can be tolerated and can safely expand cDC1s in humans. Because Flt3L has been tested in different phases of clinical trials (21, 57), the dosing used in Flt3L clinical trials can be used as a reference for the dosing of Alb-Flt3L in human trials. The safety profile from such a pilot study will allow us to design a clinical trial by combining Alb-Flt3L with chemoradiation or immune checkpoint blockage in a select group of patients with appropriate indicated pathologies to assess efficacy compared to standard-of-care approaches.

While Alb-Flt3L serves as an exciting potential therapeutic, it is not without limitations. Given the mechanism of Alb-Flt3L–mediated tumor control, it could in principle be applied nearly universally in cancer to enhance antigen-specific immunity. However, this still depends on the host having T cells capable of engaging the peptides cross-presented by Alb-Flt3L–expanded DCs. If T cell selection in the thymus did not permit those tumor antigen–specific cells from emigrating to the periphery, cross-presenting the antigens would be futile. Additionally, Alb-Flt3L depends on the tumor possessing sufficient immunodominant neoepitopes for productive T cell immunity to be engendered. This could be an issue in cancers driven by small numbers of driver mutations that lack potentially immunogenic passenger mutations. Apart from this, there is always a risk of generating immunosuppressive cells when treating with DC-tropic reagents such as Alb-Flt3L. While Alb-Flt3L–expanded DCs do present neoantigens in an inflammatory context, they can also present self-antigens or other antigens that could drive tolerogenic T cells. This can be partially combated by skewing the underlying tumor microenvironment to a more inflammatory state using chemotherapy and radiation therapy, as we do in our study. Conversely, Alb-Flt3L could present antigens that are inflammatory, but detrimental to the host such as those involved in allergy and autoimmunity. Clinical studies employing Flt3L seem to not have this issue in a substantial manner (21), but the possibility certainly exists. Regardless of these potential drawbacks, Alb-Flt3L serves as an immunotherapeutic in situ vaccination strategy with promising preclinical results that warrant further clinical development.

## Methods

### Mice.

For mouse experiments, 4- to 6-week-old C57BL/6 mice were purchased from Taconic Biosciences and housed under specific pathogen–free conditions. *Batf3^–/–^* and *FcRn^–/–^* mice were acquired from The Jackson Laboratory and bred in our in-house animal facility. Information regarding our humanized mice can be found on the Taconic website (https://www.taconic.com/mouse-model/hunog).

### Tumor cells.

TC-1 tumor cells were generated in our lab and maintained as previously described (58). MC38 cells were maintained as described in our lab (59) and were provided as a gift from Jonathan Powell (at the time of this study, Johns Hopkins University; currently, Calico Labs). B16-OVA cells were acquired as a gift from Charles Drake (at the time of this study, at Johns Hopkins University; currently, Janssen) and maintained in DMEM with 10% FBS. Cell cultures were maintained from a master bank and not allowed to reach a high passage number. Cells were always washed in excess PBS to remove any FBS prior to inoculation in mice.

### Generation of Alb-Flt3L protein constructs.

Data shown in this study utilized mouse albumin–human Flt3L. Humanized mouse experiments utilized human albumin–human Flt3L. These constructs were generated using the following methods.

For the generation of pcDNA3-Malb (mouse albumin), Malb was first amplified with PCR using cDNA template of mouse albumin (NM_000477) purchased from transOMIC technologies and a set of primers: 5′-AAATCTAGAGCCACCATGAAGTGGGTAACCTTT-3′ and 5′-TTTGAATTCGGCTAAGGCGTCTTTGCATC-3′. The amplified product was then cloned into the XhoI/EcoRI sites of the pcDNA3 vector (Invitrogen).

Next, for generation of pcDNA3-Halb (human albumin), Halb was first amplified with PCR using the cDNA template of human albumin (NM_000477) purchased from transOMIC technologies and a set of primers: 5′-AAACTCGAGGCCACCATGAAGTGGGTAACCTTT-3′ and 5′-TTTGAATTCTAAGCCTAAGGCAGCTTGAC-3′. The amplified product was then cloned into the XbaI/EcoRI sites of the pcDNA3 vector.

Next, for the generation of pcDNA3-HalbHflt3L, human Flt3L was first PCR amplified using a cDNA template of the human *Flt3L* (AAA90950.1) gene synthesized from Genscript (Piscataway, NJ) and a set of primers: 5′-TTTGAATTCACCCAGGACTGCTCCTTCCAA-3′ and 5′-AAAGGATCCTCACGAGGTCAGGAGATCGAG-3′. The amplified product was then cloned into the EcoRI/BamHI sites of pcDNA3-Halb.

To generate pcDNA3-MalbHflt3L, the human Flt3L DNA fragment was isolated from pcDNA3-HalbHflt3L and cloned into the EcoRI/AflII sites of pcDNA3-Malb.

All plasmid constructs were confirmed by DNA sequencing. HalbHflt3L and MalbHflt3L proteins were expressed using an Expi293F expression system kit (Thermo Fisher Scientific) according to the manufacturer’s instructions. Expi293F cells were transfected with pcDNA3HalbHflt3L or pcDNA3MalbHflt3L. Proteins were purified by HiTrap Albumin column (GE Healthcare Life Sciences).

### Protein labeling and in vivo trafficking.

Alb-Flt3L or Flt3L (Genscript) were labeled using Alexa Fluor 647 NHS Ester (succinimidyl ester) (Thermo Fisher Scientific) according to the manufacturer’s instructions. Proteins were purified using 7K MWCO Zebra Spin columns (Thermo Fisher Scientific). Equimolar ratios of labeled proteins were injected into TC-1 tumor–bearing mice and the mice imaged at the indicated points. Images were acquired on an IVIS spectrum optical imaging system.

### In vitro bone marrow–derived cell cultures.

To assess the differentiation of bone marrow–derived cells, femurs were collected from C57BL/6, *FcRn^–/–^*, or *Batf3^–/–^* mice, and cells were flushed. Cells were washed extensively, RBCs lysed, and plated at 1 million cells per well in a 6-well plate. Flt3L (100 ng/mL), Alb-Flt3L (500 ng/mL), or GM-CSF (20 ng/mL) was added to cells. New cytokine-supplemented growth media were added on days 3 and 5 after culture. Cells were collected on day 7 for analysis by flow cytometry.

### Half-life determination ELISA.

Fifty micrograms of Alb-Flt3L or 10 μg of Flt3L (Genscript) (equimolar ratio) was injected into C57BL/6 mice. Blood was collected at the indicated time points. Serum was used for ELISA. Anti–human Flt3L and anti–human Flt3L–biotin antibody pairs were purchased (R&D Systems, BAF308 and MAB308). Anti–human Flt3L was coated onto ImmunoGrade plates (Genesee). Serum from mice was diluted in 10% FBS/PBS, added to plates, and incubated for 2 hours. Plates were then washed and incubated with anti–human Flt3L–biotin antibody plus Streptavidin-HRP (Thermo Fisher Scientific). After the final wash, plates were developed using TMB Ultra substrate (Thermo Fisher Scientific) and stopped using 2 M H_2_SO_4_ before OD was measured using a spectrophotometer at 450 nm and 590 nm.

### Sample preparation for flow cytometry.

For PBMC analysis, blood was collected into EDTA-coated tubes by submandibular bleeding. RBCs were lysed by addition of excess RBS lysis buffer twice (Cell Signaling Technology) followed by extensive washing in 0.5% BSA/PBS (FACS buffer). For spleen or tumor cell analysis, tissue was collected from mice into FACS buffer with magnesium and calcium. After mincing with scissors into approximately 2-mm pieces, tissue digestion enzymes, including collagenase I, collagenase IV, and DNase I were added to samples and incubated for 20 minutes at 37°C. Excess 10% FBS/RPMI was then added to the samples to quench the reaction. After centrifugation and extensive washing, tumor samples were purified by Ficoll gradient. Briefly, cells were resuspended in 10% FBS/RPMI and loaded onto Ficoll-Paque Plus (GE Healthcare Life Sciences). Tubes were centrifuged for 20 minutes with low acceleration and no brake. The Ficoll-RPMI interface was collected and samples were then counted, plated at equal cell numbers, and prepared for flow cytometry as described below.

### Flow cytometry and cell sorting.

For all experiments utilizing flow cytometry, single-cell suspensions were prepared and extensively filtered prior to acquisition. Single-staining controls consisting of UltraComp beads (Thermo Fisher Scientific) were used to set the compensation matrix for each experiment. FMO and isotype staining controls were used to set gating and determine nonspecific binding. Prior to antibody staining, Zombie Aqua Live/Dead (BioLegend) or ViaKrome 808 Fixable Viability Dye (Beckman Coulter) was used for dead cell discrimination according to the manufacturers’ instructions. Fc Block was used prior to antibody staining. Antibody and tetramer dilutions were determined through titration. All antibody mixes were stained for at least 30 minutes at 4°C. Samples were all acquired on either a 13-color Beckman Coulter CytoFLEX S at Johns Hopkins or 21-color Beckman Coulter CytoFLEX LX flow cytometer at the NIH National Eye Institute. For cell sorting, a Beckman Coulter MoFlo XDP sorter was used at the Johns Hopkins Bloomberg School of Public Health core facility. A list of all antibodies used in this work can be found in Supplemental Table 1.

### Intracellular staining.

For staining intracellular cytokines, samples were incubated with PMA/ionomycin cell stimulation cocktail and Brefeldin A with Monensin Golgi Plug (Thermo Fisher Scientific) for 4 hours at 37°C. Cells were then collected and prepared as described in *Flow cytometry and cell sorting*. At the end of extracellular staining, cells were permeabilized using an eBioscience Foxp3/Transcription Factor Staining Buffer Set (Thermo Fisher Scientific) and stained for intracellular cytokines or Ki-67 as appropriate.

### Tumor experiments.

Tumor cells for inoculation were all prepared from cultures of at least 90% viability as determined by trypan blue staining. Cells were prepared in PBS for implantation subcutaneously in the abdomen or lower flank for experiments where radiation therapy was administered. Tumor growth was monitored by digital caliper reading and tumor volume was calculated using the formula length × length × width × 0.5. Experimental endpoints were determined once tumors were greater than 2 cm in diameter or when any loss in body weight was observed as per our approved animal protocols. For experiments where IFNAR1 blockade or pDC depletion was performed, mice were administered 500 μg of anti-IFNAR1 or 200 μg of anti–PDCA-1 three times per week for a total of 4 weeks. For all treatments with Alb-Flt3L or Flt3L, 50 μg of Alb-Flt3L in 100 μL of PBS or 10 μg of Flt3L in 100 μL of PBS was administered intravenously through retroorbital injection unless stated otherwise.

### Radiation therapy and chemotherapy.

For the delivery of radiation therapy, the Small Animal Radiation Research Platform (SARRP) was used. Following anesthesia with isoflurane, tumors were imaged by cone beam computed tomography (CBCT) for dose calculation and targeting. Radiation (10 Gy) was then administered to each mouse as a single fraction. For the administration of chemotherapy, *cis*-dichlorodiammine platinum(II) (cisplatin) was solubilized in PBS at 1 mg/mL. One hundred microliters of this solution (100 μg) was then administered to mice intraperitoneally.

### Next-generation sequencing.

For TCR sequencing experiments, tumors were harvested, digested, and single-cell suspensions were prepared as described above. Samples were then stained with lineage markers and anti-CD8 and ADPGK tetramer as appropriate. CD8^+^ or CD8^+^ ADPGK-tetramer^+^ cells were sorted as described above. T cells were submitted to Adaptive Biotechnologies for ImmunoSeq and analyzed using immunoSEQ Analyzer. For scRNA-seq, single cells from spleens were collected from humanized mice treated with Alb-Flt3L or vehicle control from donor-matched mice. Human cells were isolated using the EasySep Mouse/Human Chimera Isolation Kit (STEMCELL Technologies). Samples were then submitted to the Johns Hopkins Genetic Resources Core Facility Cell Center for capture and library preparation using the 10× Genomics Chromium platform (approximately 6000 cells per group). Following a small MiSeq to confirm library quality, a NovaSeq SP100 run was performed. Analysis was run using Loupe Browser (https://www.10xgenomics.com/support/software/loupe-browser) and the R package Seurat v3 (https://satijalab.org/seurat/).

### Statistics.

All data are expressed as mean ± SEM. Results for flow cytometric analysis and tumor treatment experiments were evaluated by analysis of variance (1-way ANOVA) and the Tukey-Kramer multiple-comparison test where appropriate. Comparisons between individual data points were made using Student’s *t* tests. Survival analysis was performed using Kaplan-Meier survival curves and log-rank tests. All *P* values less than 0.05 were considered significant, and those that are not are listed as not significant (NS): *P < 0.05, **P < 0.01, ***P < 0.001, ****P < 0.0001. All statistical calculations were performed in GraphPad Prism 9.

### Study approval.

The housing and handling of mice followed guidelines established by Johns Hopkins Medical Institutions Animal Care and Use Committee and the NIH *Guide for the Care and Use of Laboratory Animals* (National Academies Press, 2011). Animals were monitored daily for infection and other illnesses by trained animal technicians. Only trained laboratory personnel and animal technicians were allowed to handle laboratory animals. All individuals handling mice were registered in protocols at the Johns Hopkins Animal Care and Use Committee.

### Data availability.

Raw data used to generate plots in the manuscript are available as an additional supplemental Supporting Data Values file. The scRNA-seq data have been deposited in the Johns Hopkins University Data Services (https://archive.data.jhu.edu/) and can be accessed via https://doi.org/10.7281/T1/NQLGNR Any additional data requests or questions about data analysis can be directed to the first author or corresponding author.

## Author contributions

B Lam and CFH conceived of and designed the study. B Lam, YJK, JL, SHT, CH, B Lee, EV, YCT, RV, HFT, DX, and STP conducted experiments. B Lam, YJK, JL, SHT, CH, B Lee, EV, YCT, RV, HFT, DX, and STP analyzed and interpreted data. B Lam, YJK, JL, and CFH wrote and reviewed the manuscript. TCW and CFH supervised the study.

## Supplementary Material

Supplemental data

Supplemental table 1

Supporting data values

## Version 1 11/02/2023

In-Press Preview

## Version 2 01/02/2024

Electronic publication
