# Supplementary material for: In situ vaccination via tissue-targeted cDC1 expansion enhances the immunogenicity of chemoradiation and immunotherapy
Source: J Clin Invest. 2024 Jan 2;134(1):e171621. doi: 10.1172/JCI171621 (PMC10760964; doi:10.1172/JCI171621)
Supplement: Supplemental data [file jci-134-171621-s262.pdf]

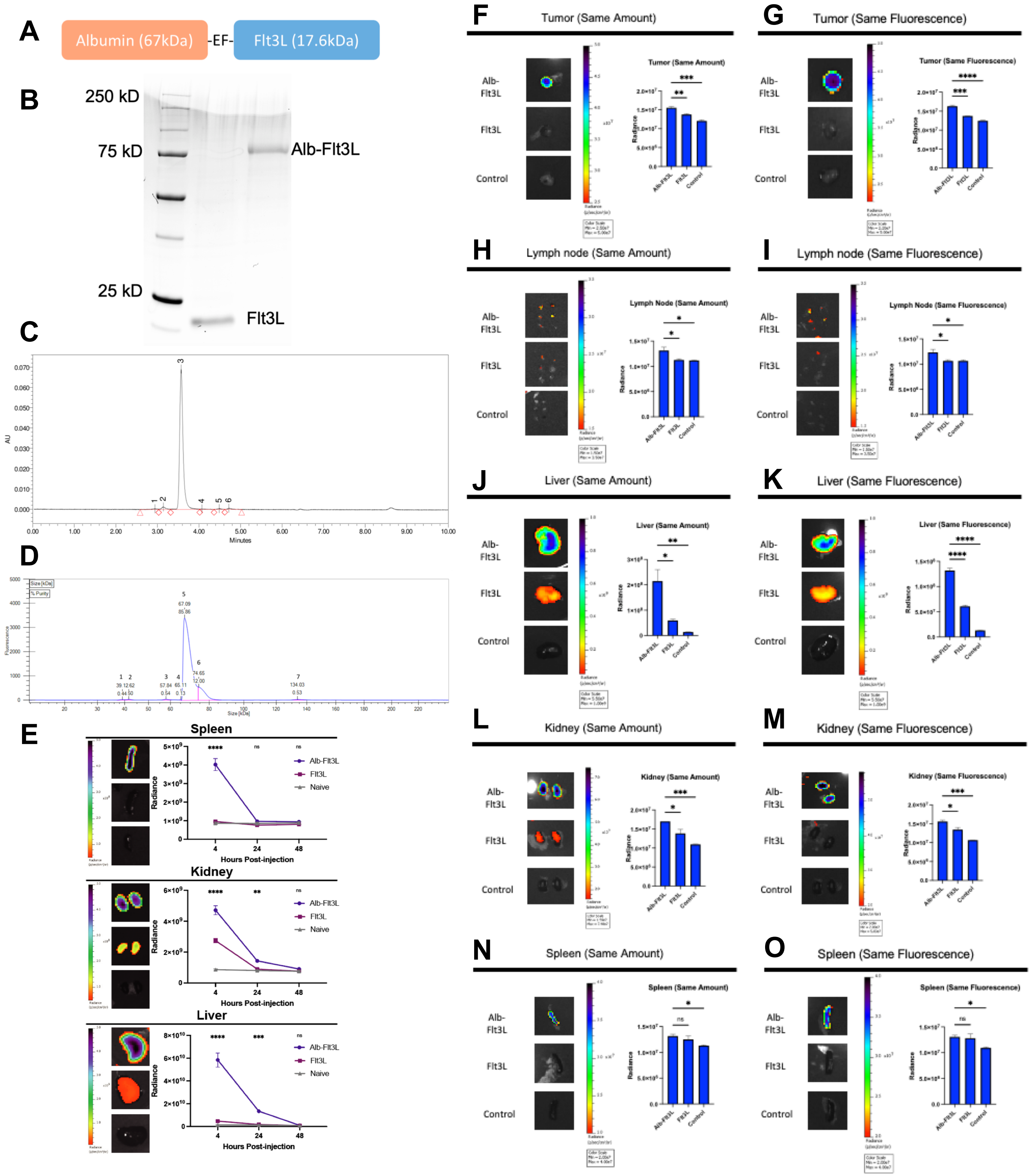

Supplementary Figure 1. Alb-Flt3L construct and tissue targeting. (a) Alb-Flt3L schematic. **(b)** SDS-Page of Flt3L and Alb-Flt3L. **(c)** UPLC and **(d)** native page analysis of Alb-Flt3L. **(e)** Alb-Flt3L trafficking to the spleen, kidney, and liver of mice. Mice were injected with either the same amount of protein, 20ug, or a normalized amount of protein based on fluorescent intensity and imaged at 18 hours post injection for trafficking to the **(f, g)** tumor, **(h, i)** lymph nodes, **(j, k)** liver, **(l, m)** kidney, and **(n, o)** spleen. **Data disclosure: Control tissues used to generate F-O are from the same animals, as these were from untreated mice used to assess background fluorescence levels. Significance determined using ANOVA.**

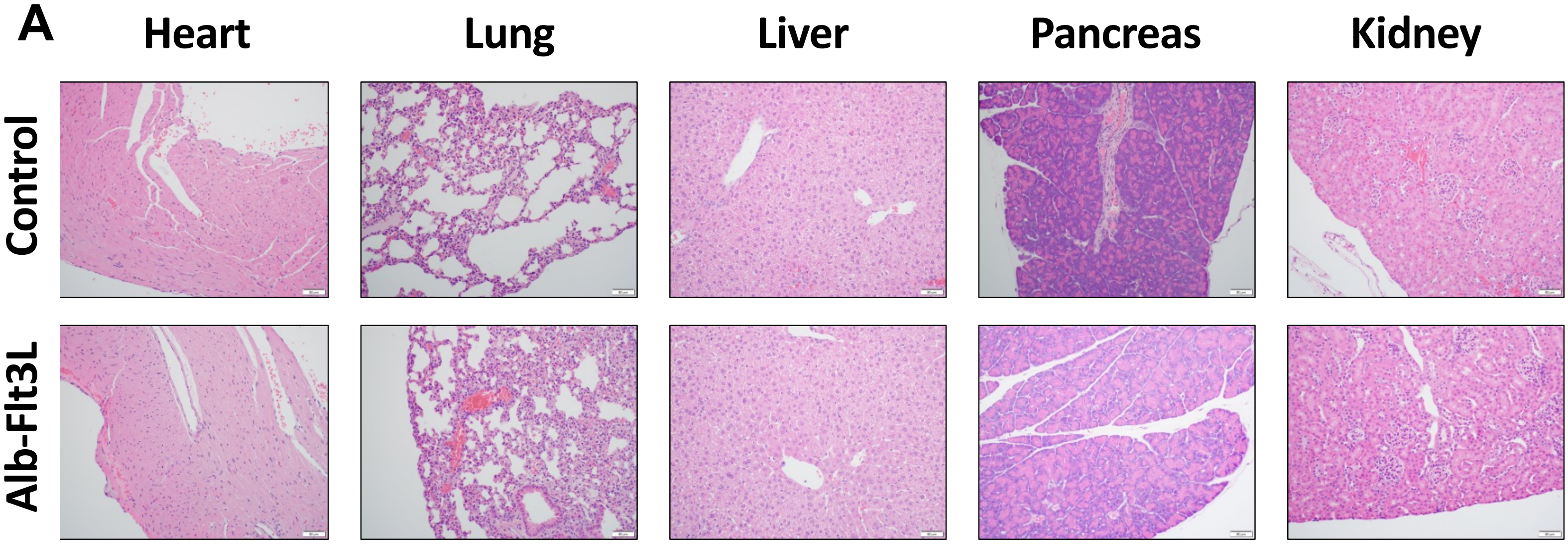

Supplementary Figure 2. H&E staining of major organs in C57BL/6 mice treated with Alb-Flt3L treatment. (a) C57BL/6 mice were administered either Alb-Flt3L (100 ug) or vehicle control via i.p. injection for 12 days. Heart, lung, liver, pancreas, and kidney tissues were harvested and stored in 10% formalin prior to paraffin-fixed tissue embedding and sectioning. Representative images of H&E stain of major organs from different treatment groups (20x). Images were reviewed by two separate board-certified pathologists and indicated that no significant differences were present.

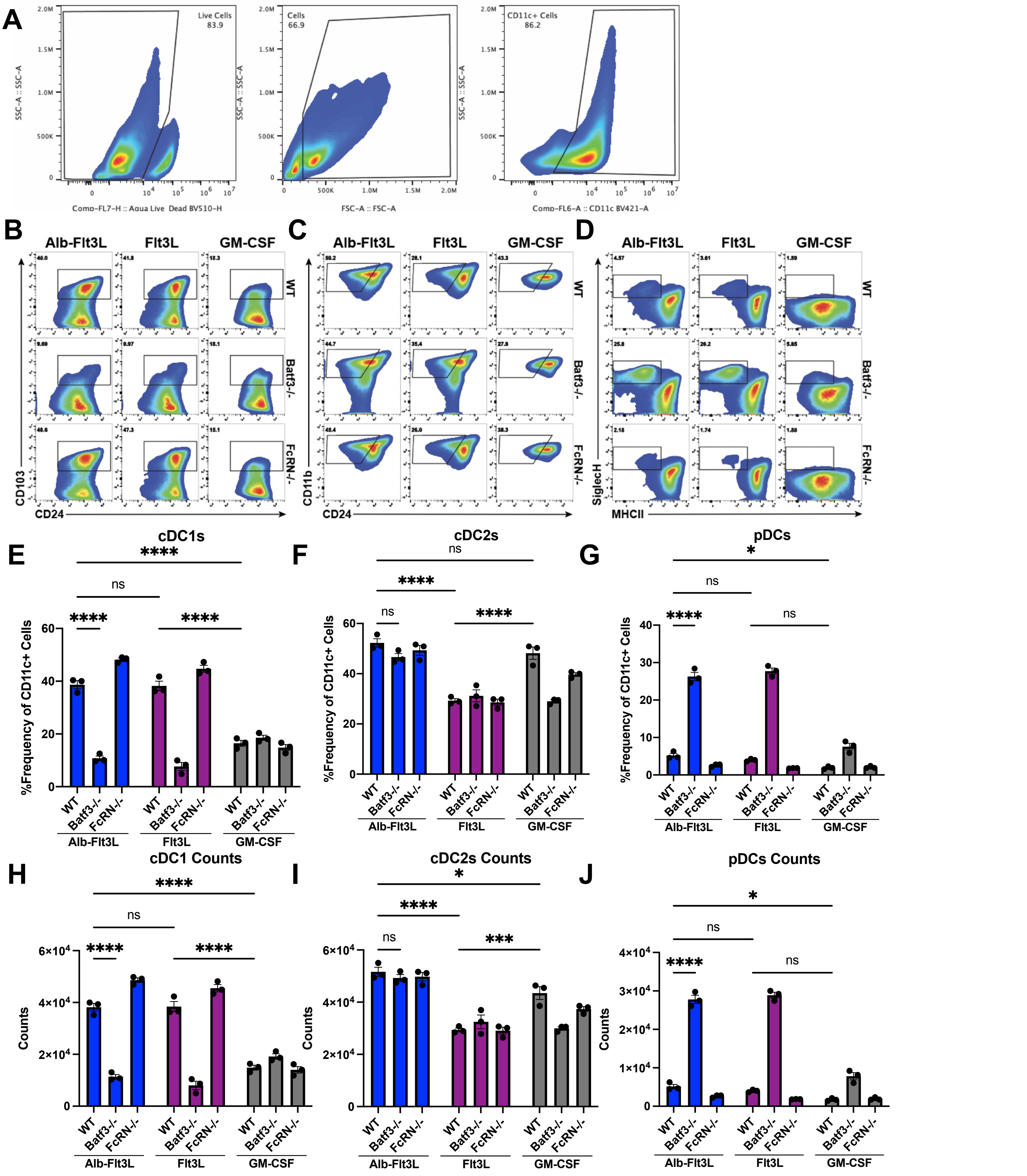

Supplementary Figure 3. Biological activity and *in vitro* expansion of DCs by Alb-Flt3L. Bone marrow progenitor cells from WT, Batf3-/-, or FcRN-/- mice were cultured with either Alb-Flt3L, Flt3L, or GM-CSF. 7 days later, cells were collected and DCs analyzed by flow cytometry. **(a)** Gating used to identify DCs. Representative gating of **(b)** cDC1s, **(c)** cDC2s, or **(d)** pDCs. Frequency of **(e)** cDC1s, **(f)** cDC2s, or **(g)** pDCs following culture of cells from the indicated mice with the indicated condition (n=3). Counts of **(h)** cDC1s, **(i)** cDC2s, or **(j)** pDCs following culture of cells from the indicated mice with the indicated condition (n=3). Significance determined using ANOVA.

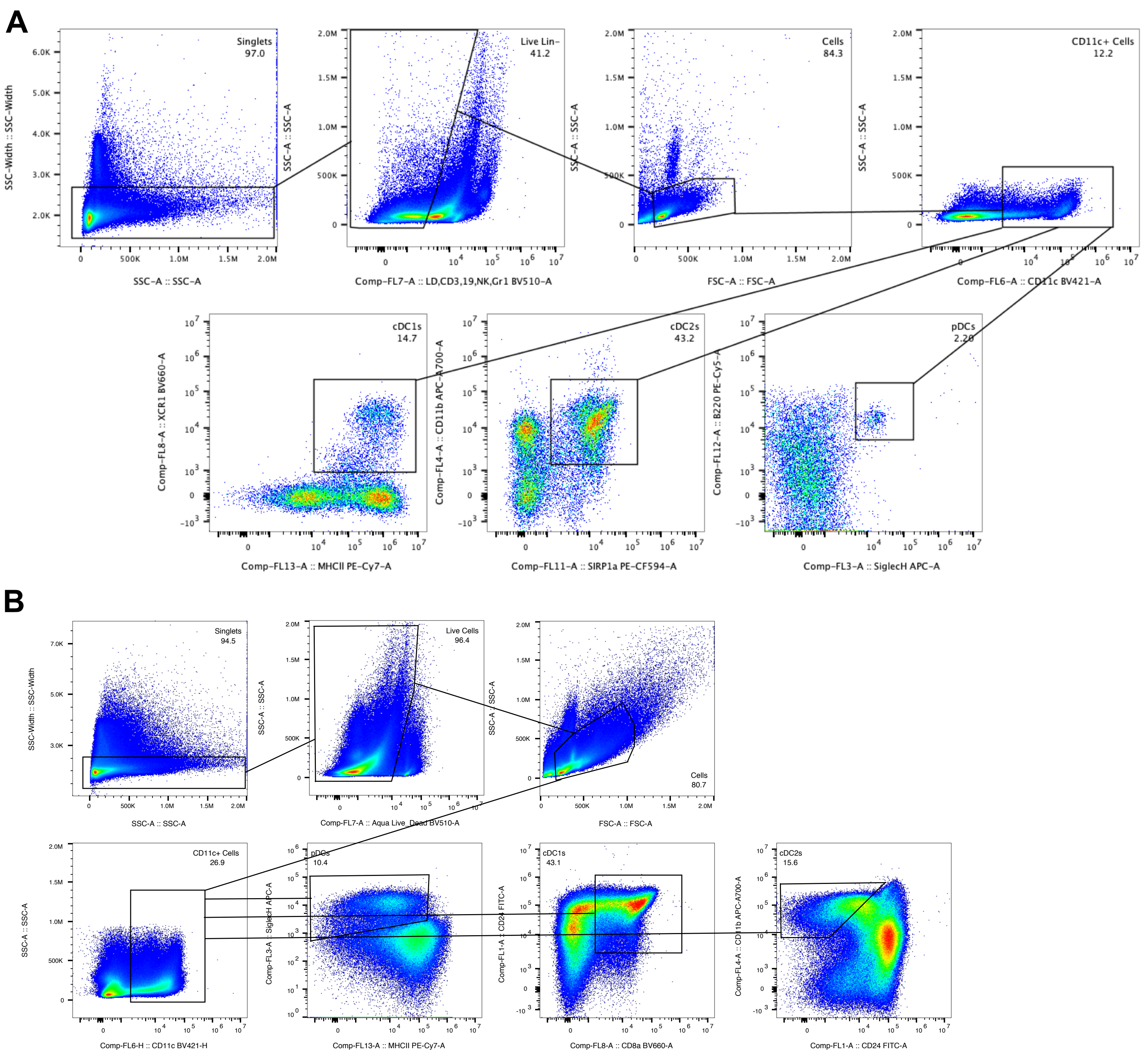

Supplementary Figure 4. Expansion of DCs by Alb-Flt3L *in vivo* gating strategy. (a) Gating strategy used to identify DCs in kinetic of DC expansion following Alb-Flt3L treatment. **(b)** Gating strategy used to identify DCs in mice treated with Alb-Flt3L.

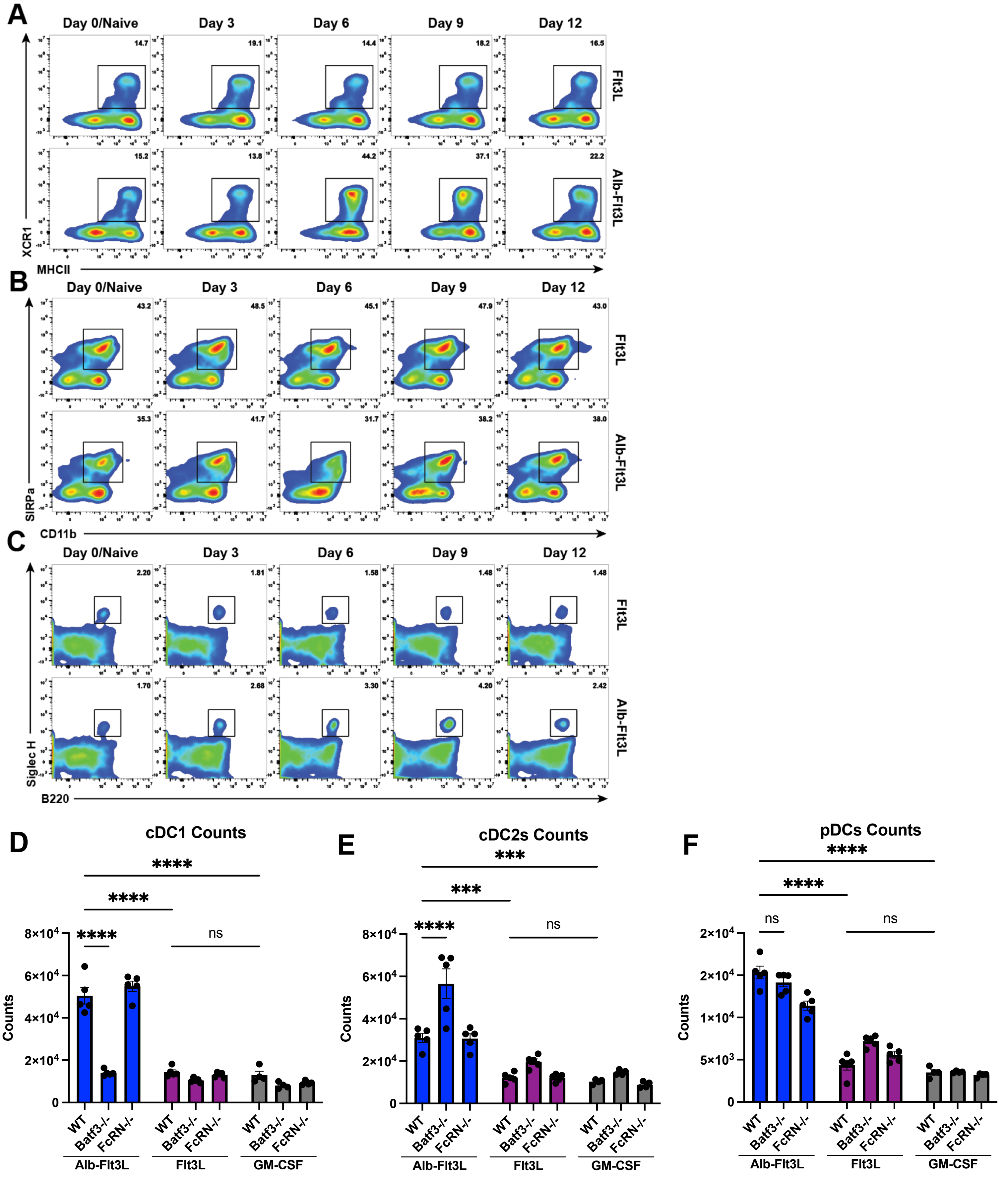

Supplementary Figure 5. Expansion of DCs by Alb-Flt3L *in vivo*. Representative dot plots of kinetic expansion of (a) cDC1s, (b) cDC2s, and (c) pDCs following Alb-Flt3L treatment. Counts of **(d)** cDC1s, **(e)** cDC2s, or **(f)** pDCs in splenocytes of all CD11c+ cells following treatment of the indicated mice with the indicated condition (n=5). Significance determined using ANOVA.

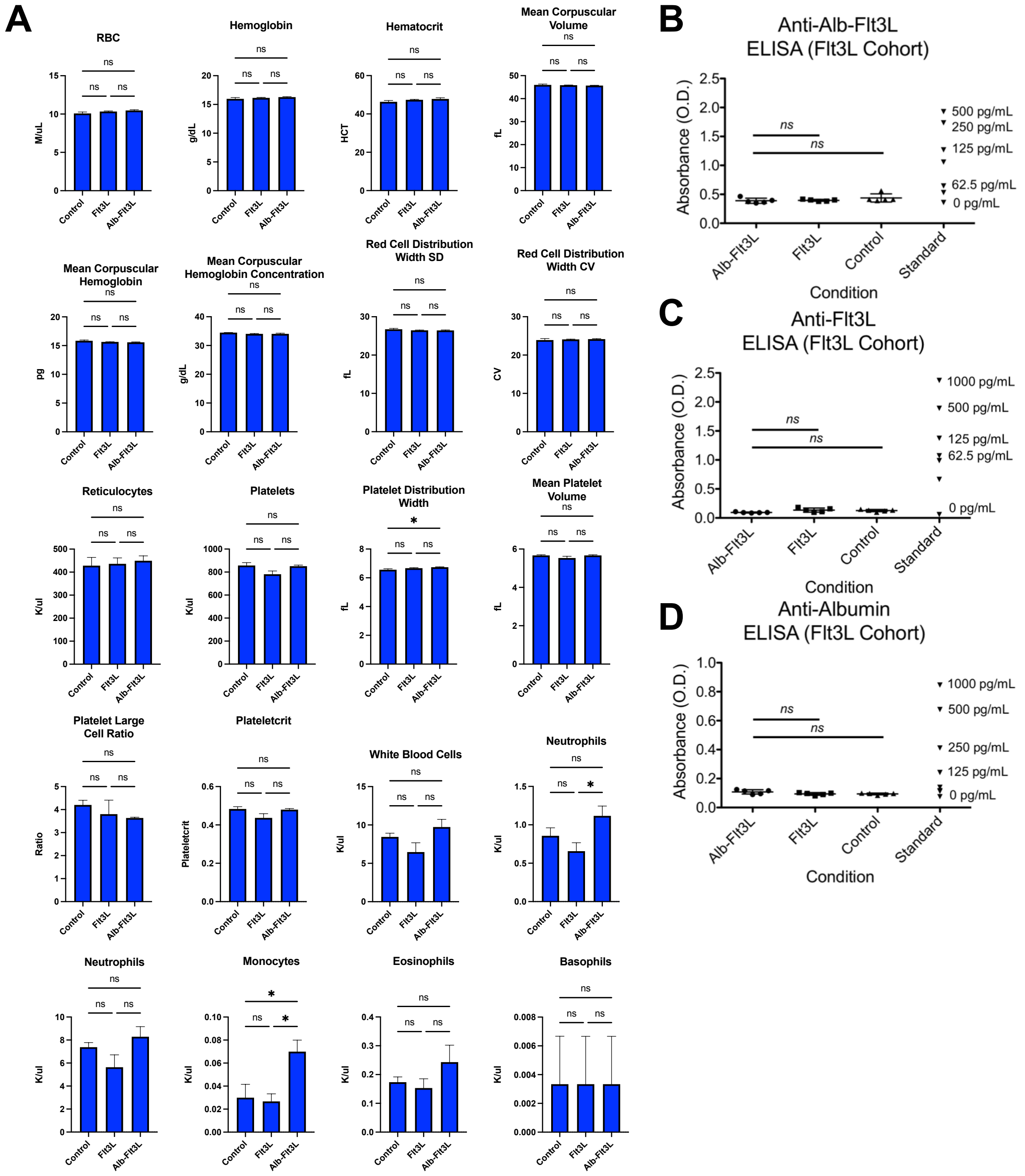

Supplementary Figure 6. Alb-Flt3L safety. (a) Complete blood count of mice treated with vehicle, Alb-Flt3L or Flt3L after 5 days. **(b)** Anti-Alb-Flt3L, **(c)** Anti-Flt3L, or **(d)** anti-Albumin antibodies after 3 treatments with vehicle, Alb-Flt3L or Flt3L. Significance determined using ANOVA.

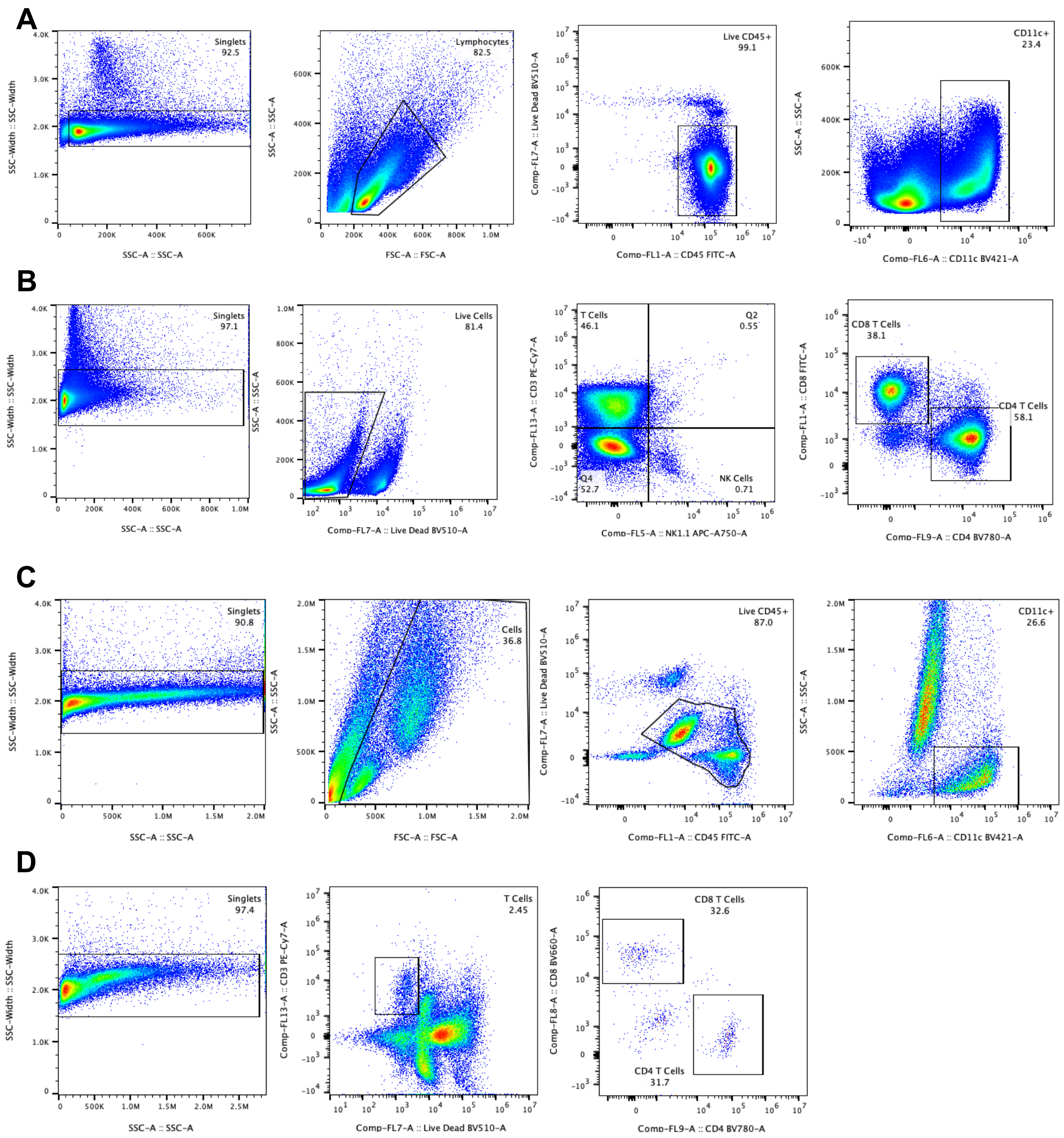

Supplementary Figure 7. Murine gating strategy. Representative flow strategy used to identify DCs and T cells respectively from the lymph node **(a,b)** and tumor **(c,d)** of B16-OVA tumor bearing mice.

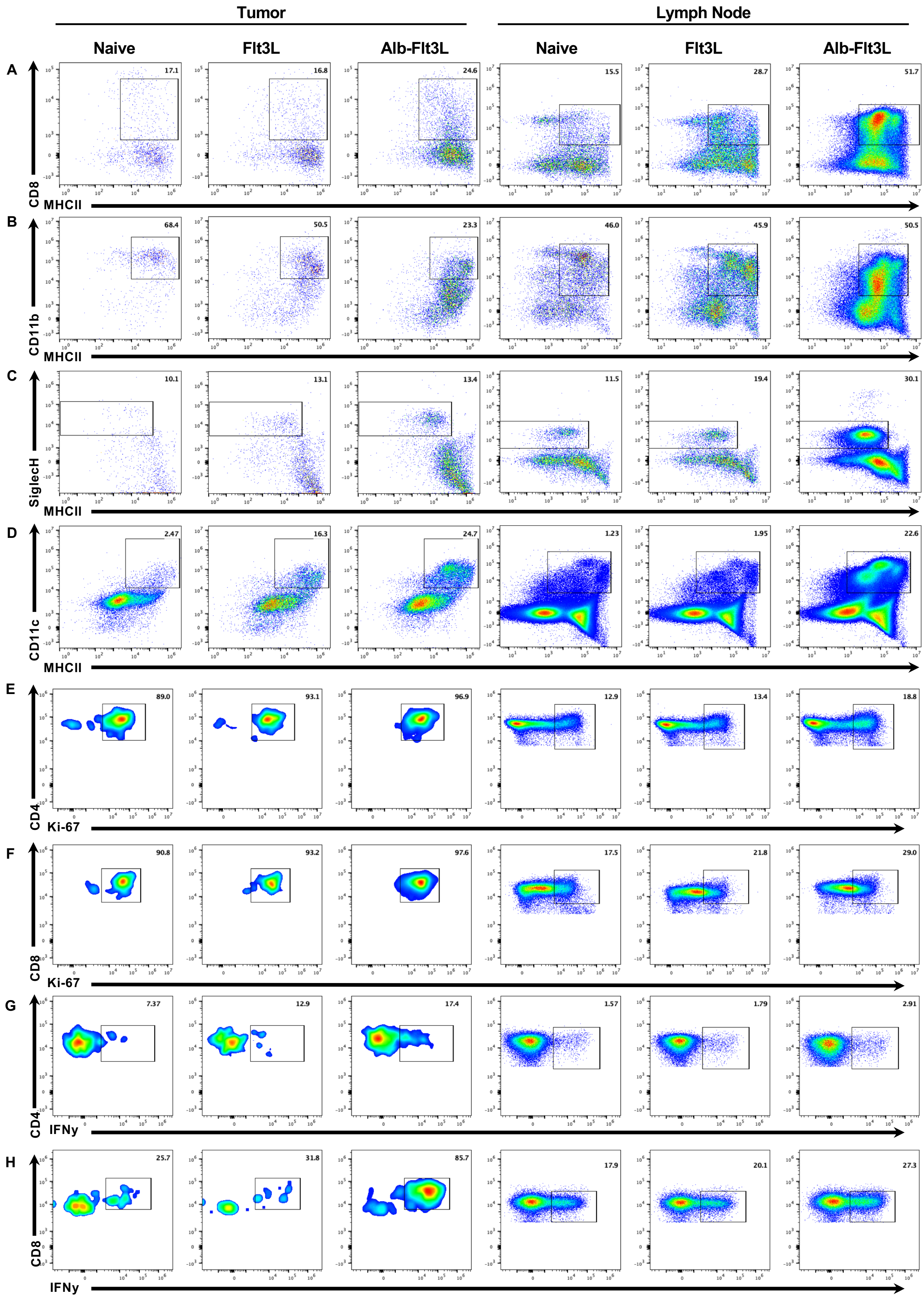

Supplementary Figure 8. Immune response of mice treated with Alb-Flt3L, Flt3L, or vehicle control. Representative flow gating of a) cDC1s, b) cDC2s, c) pDCs, d) DCs e) CD4+ Ki-67+, f) CD8+ Ki-67+, g) CD4+ IFNy+, h) CD8+ IFNy+ in the tumor and lymph node following treatment.

A

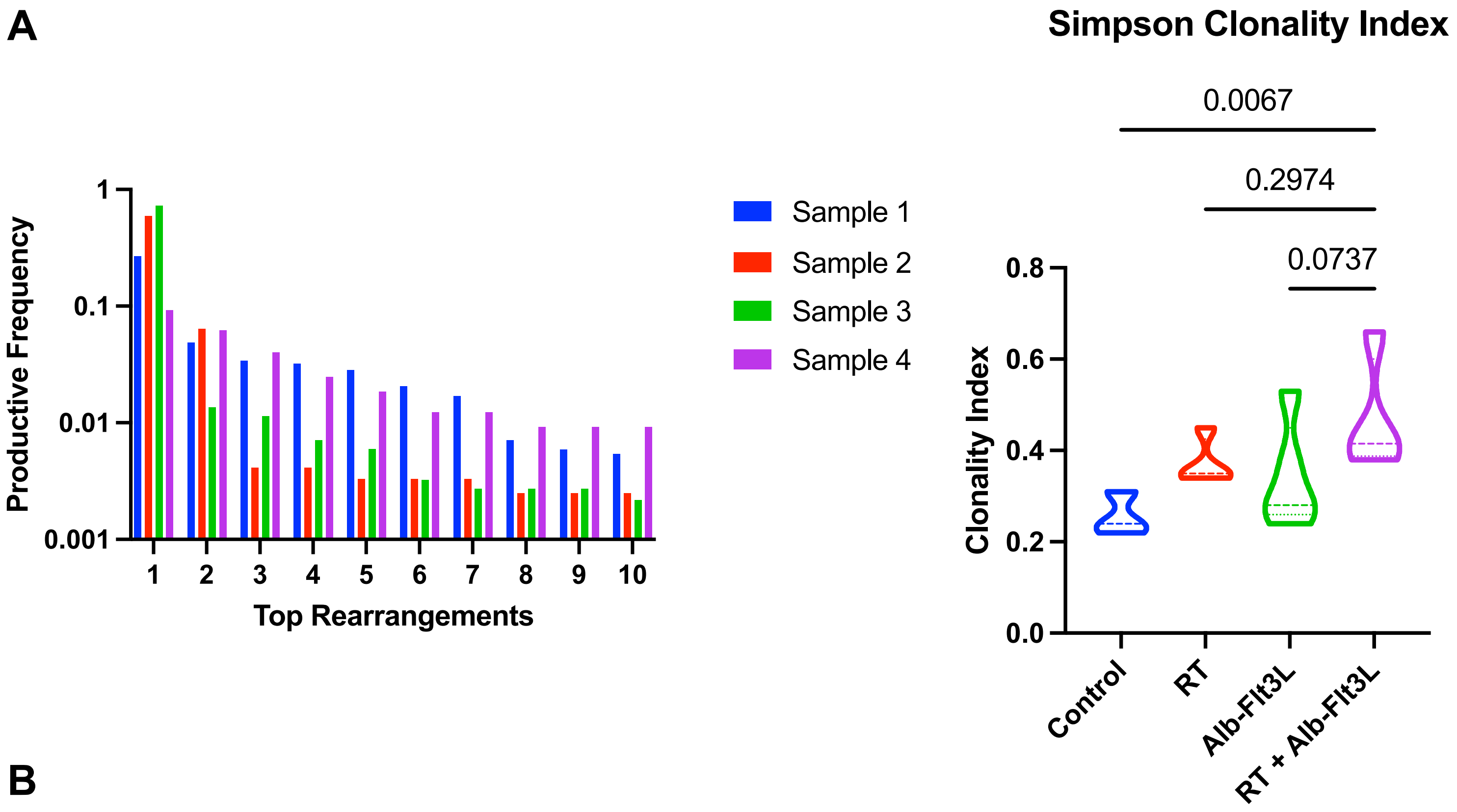

B

Sample	Total Templates	Productive Templates	Total Rearrangements	Productive Rearrangements	Max Productive Frequency
1	9167	6088	3220	1997	0.27
2	2887	1208	466	292	0.59
3	3431	1845	504	294	0.73
4	509	324	321	179	0.09

Supplementary Figure 9. TCR-sequencing of CD8+ or ADPGK Tetramer+ cells in mice treated with Alb-Flt3L + RT. (a) Top rearrangements and productive frequency of ADPGK tetramer+ cells collected from tumors of mice treated with Alb-Flt3L + RT. **(b)** Summary results of ADPGK tetramer+ cells collected from tumors of mice treated with Alb-Flt3L + RT. **(c)** Simpson clonality index determined from sorted tumor-infiltrating cells in the indicated treatment groups. Significance determined using ANOVA.

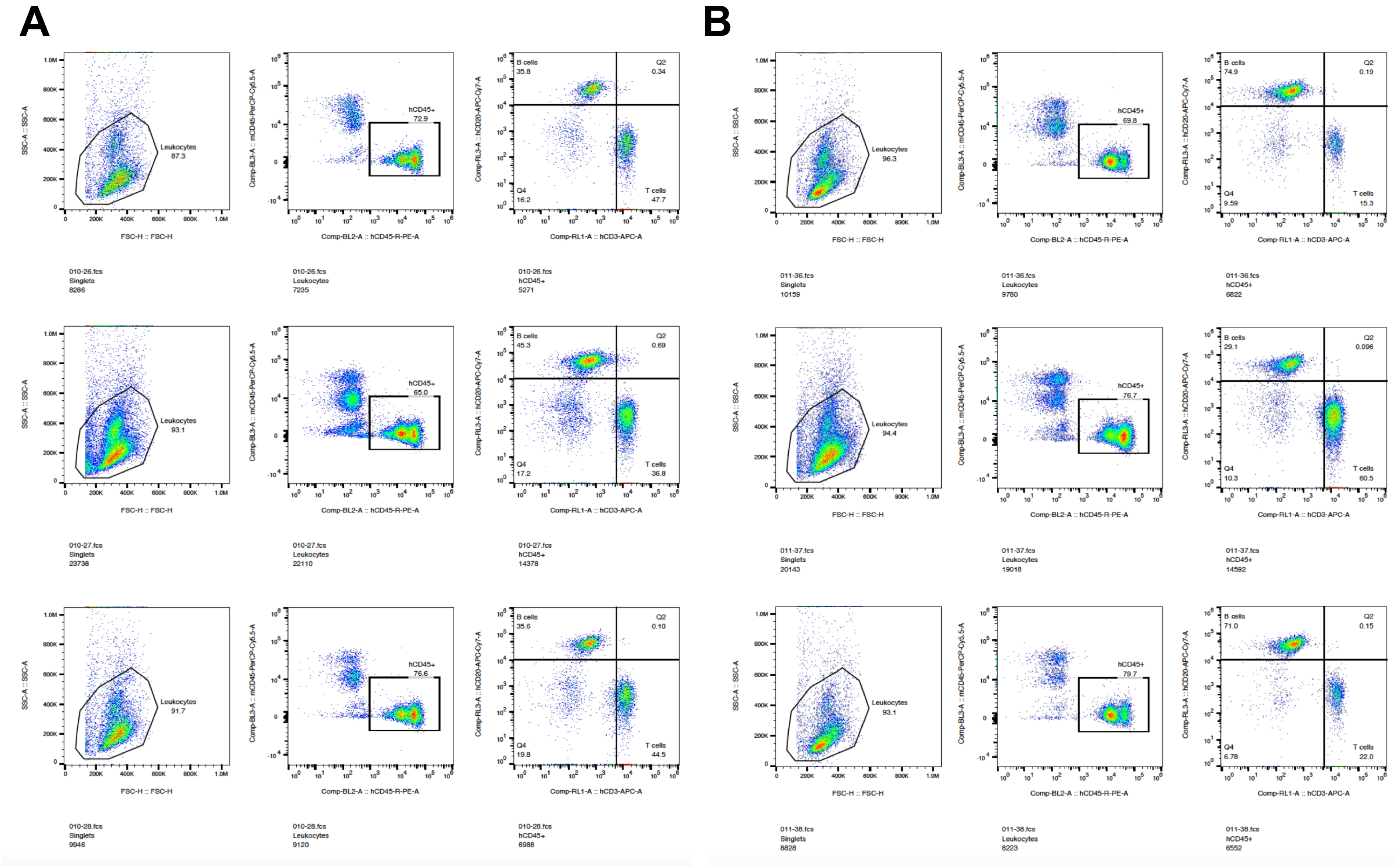

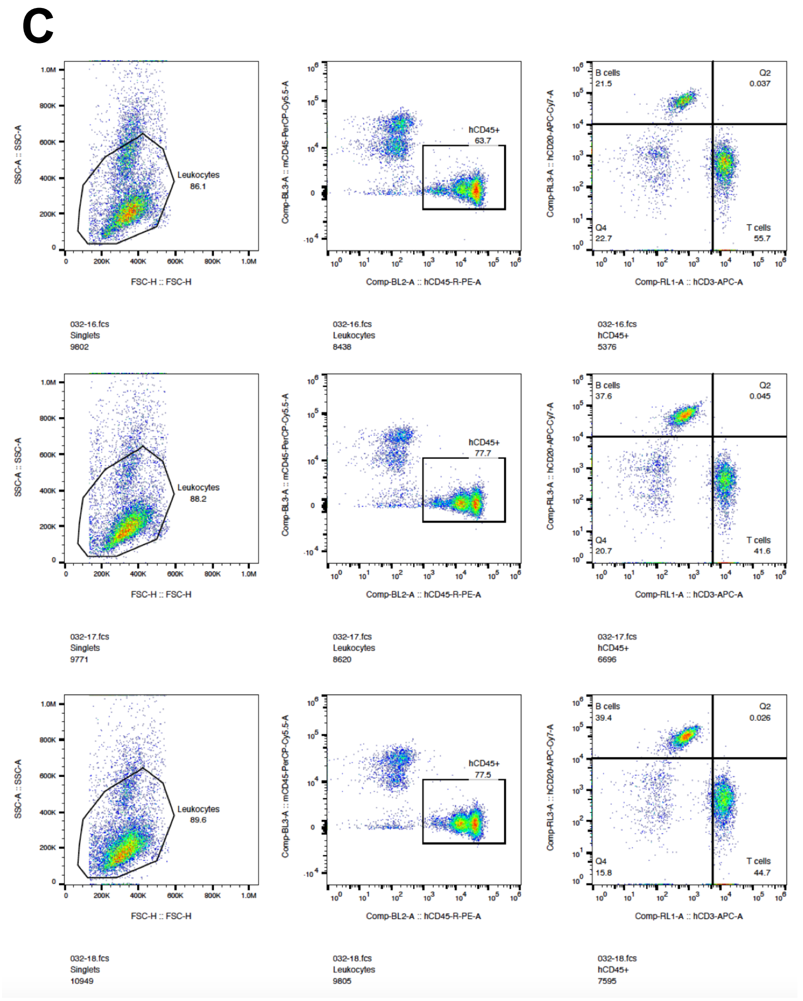

Supplementary Figure 10. Quality control metrics for humanized mice. Flow cytometry staining for the identification of the indicated human immune cell populations in humanized mice pre-treatment. Data generated by Taconic Biosciences. Representative mouse showed from each human donor lot used **(a-c).**

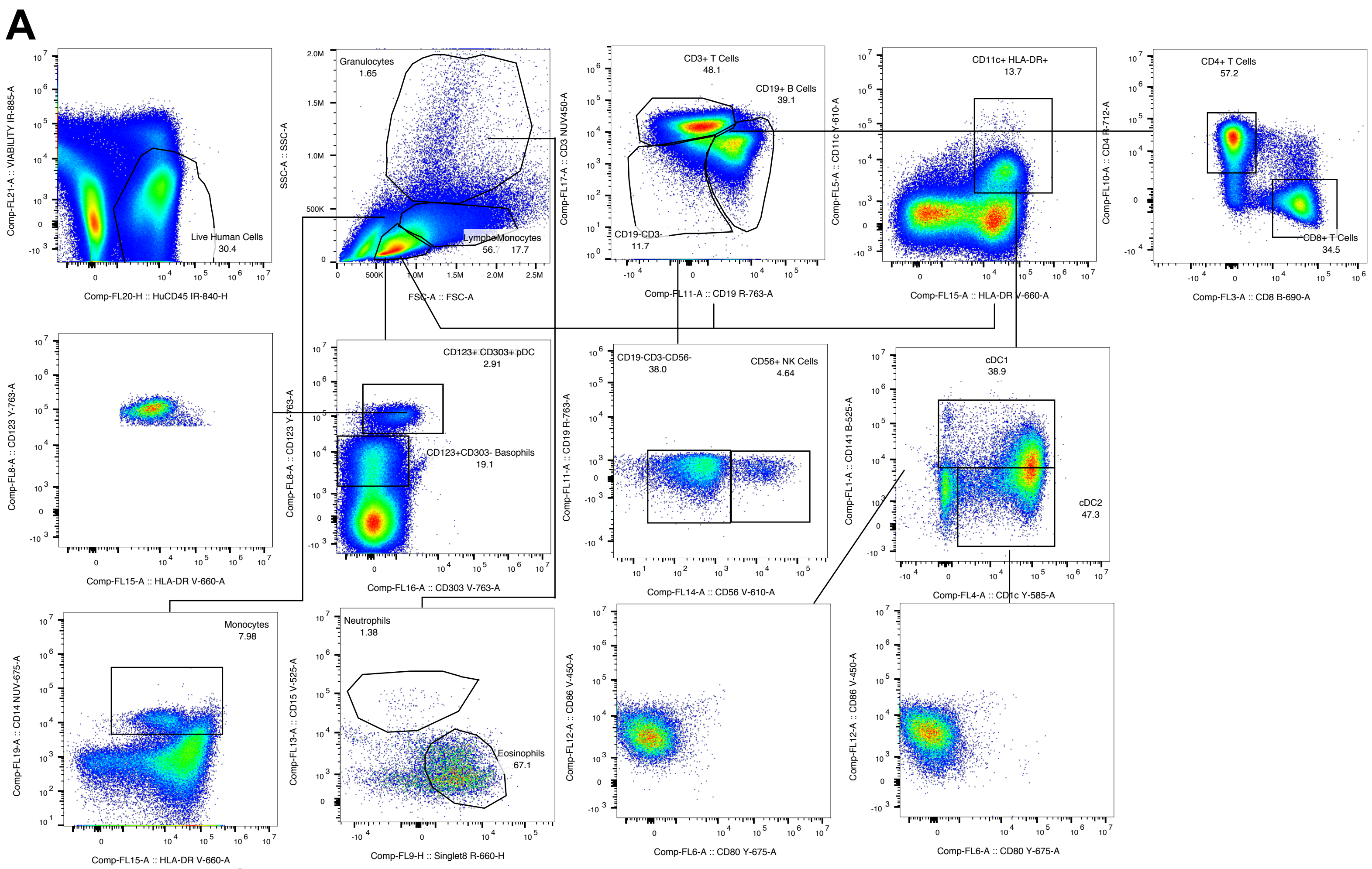

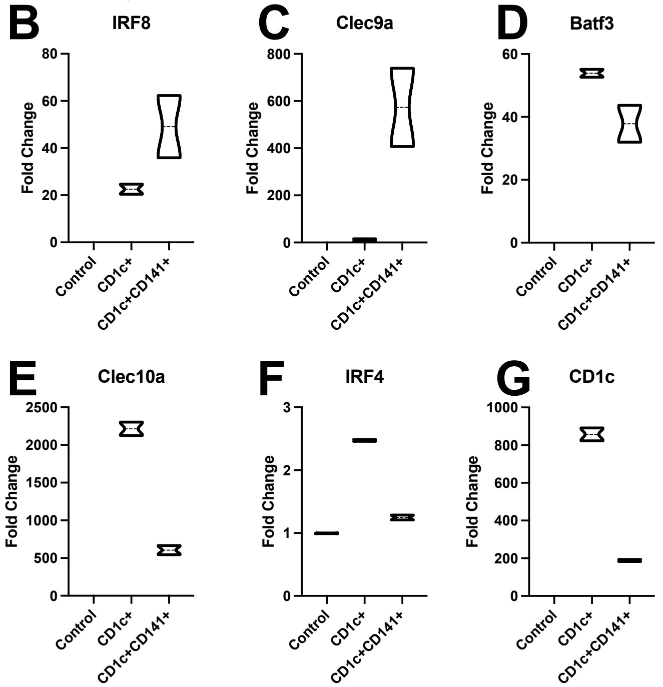

Supplementary Figure 11. Expansion of human DCs by Alb-Flt3L. (A) Representative flow gating strategy for identification of the indicated human immune cell populations in humanized mice treated with Flt3L, Alb-Flt3L, or control. This example is from mice treated with Alb-Flt3L **(B-G)** qPCR data confirming lineage-defining protein or transcription factor expression in cDC1s (CD1c+CD141+) and cDC2s (CD1c+CD141-) sorted from spleens of mice treated with Alb-Flt3L. Data was first normalized to housekeeping gene (18S), and then fold change was calculated compared to control. Counts of **(h)** DCs, **(i)** cDC1s, **(j)** cDC2s, and **(k)** pDCs in mice treated with the indicated conditions. Control is from CD11c- cells. Significance determined using ANOVA.

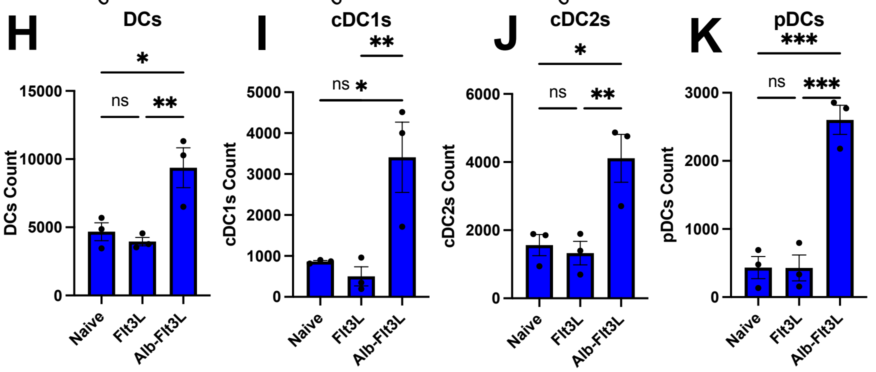

Reagent	Catalog Number	Company
iFluor™ 860	1408	ATT Bioquest
Zombie AquaTM Fixable Viability Kit	423102	BioLegend
BB515 Mouse Anti-Human CD141	565084	BD
Alexa Fluor® 594 anti-mouse CD45	103144	Biolegend
PerCP/Cyanine5.5 anti-human CD8	565310	BD
PE anti-human CD1c	564900	BD
PE/Dazzle™ 594 anti-human CD11c	301642	Biolegend
PE/Cy5 anti-human CD80	305210	Biolegend
PE/Cy7 anti-human CD123	306010	Biolegend
APC anti-human Siglec-8 Antibody	347106	Biolegend
APC-R700 Anti-Human CD4	564975	BD
APC/Fire™ 750 anti-human CD19	363030	Biolegend
Brilliant Violet 421™ anti-human CD86	305426	Biolegend
Brilliant Violet 510™ anti-human CD15 (SSEA-1) Antibody	323028	Biolegend
Brilliant Violet 605™ anti-human CD56	318334	Biolegend
Brilliant Violet 650™ anti-human HLA-DR	307650	Biolegend
Brilliant Violet 785™ anti-human CD303	354222	Biolegend
BUV395 Anti-Human CD3	563546	BD
BUV661 Anti-Human CD14	741603	BD
BUV805 Anti-Human CD45	612891	BD
Alexa Fluor® 488 anti-mouse CD24	101816	Biolegend
PE anti-mouse CD370 (CLEC9A, DNGR1)	143504	Biolegend
Alexa Fluor® 594 anti-mouse CD80	104754	Biolegend
PE/Cyanine5 anti-mouse CD40	124618	Biolegend
PE/Cyanine7 anti-mouse I-A/I-E	107630	Biolegend
APC anti-mouse Siglec H	129612	Biolegend
APC-R700 Rat Anti-CD11b	564985	BD
APC/Cyanine7 anti-mouse CD103	121432	Biolegend
Brilliant Violet 421™ anti-mouse CD11c	117330	Biolegend
Brilliant Violet 650™ anti-mouse CD8a	100742	Biolegend
Brilliant Violet 785™ anti-mouse CD86	105043	Biolegend
Alexa Fluor® 594 anti-mouse/human CD44	103054	Biolegend
APC/Cyanine7 anti-mouse CD3	100222	Biolegend
APC anti-mouse CD69	104514	Biolegend
APC-R700 Rat Anti-Mouse CD62L	565159	BD
Alexa Fluor® 488 anti-mouse Ki-67	652417	Biolegend
Brilliant Violet 785™ anti-mouse CD4	100453	Biolegend
APC anti-mouse IFN-γ	505810	Biolegend
Brilliant Violet 650™ anti-mouse TNF-α	506333	Biolegend
PE/Cyanine5 anti-mouse/human CD45R/B220	103209	Biolegend
Brilliant Violet 650™ anti-mouse/rat XCR1	148220	Biolegend
Brilliant Violet 785™ anti-mouse CD45	103149	Biolegend
Brilliant Violet 510™ anti-mouse CD3	100233	Biolegend
Brilliant Violet 510™ anti-mouse CD19	115545	Biolegend
Brilliant Violet 510™ anti-mouse NK-1.1	108737	Biolegend
Brilliant Violet 510™ anti-mouse Ly-6G/Ly-6C (Gr-1)	108437	Biolegend
Anti-IFNAR-1	BE0241	BioXCell
Anti-CD317/PDCA-1	BE0311	BioXCell

Supplementary Table 1. Flow Cytometry antibody list. List of reagents used for flow cytometry.
